# Exploring the microbiome of oral epithelial dysplasia as a predictor of malignant progression

**DOI:** 10.1186/s12903-023-02911-5

**Published:** 2023-04-06

**Authors:** Robyn J. Wright, Michelle E. Pewarchuk, Erin A. Marshall, Benjamin Murrary, Miriam P. Rosin, Denise M. Laronde, Lewei Zhang, Wan L. Lam, Morgan G. I. Langille, Leigha D. Rock

**Affiliations:** 1 Department of Pharmacology, Dalhousie University, Halifax, Canada; 2 Department of Integrative Oncology, British Columbia Cancer Research Centre, Vancouver, Canada; 3 Department of Cancer Control Research, British Columbia Cancer Research Centre, Vancouver, Canada; 4 Department of Biomedical Physiology and Kinesiology, Simon Fraser University, Burnaby, Canada; 5 Faculty of Dentistry, University of British Columbia, Vancouver, Canada; 6 Oral Biopsy Service, Vancouver General Hospital, Vancouver, Canada; 7 Beatrice Hunter Cancer Research Institute, Halifax, Canada; 8 Faculty of Dentistry, Dalhousie University, Halifax, Canada; 9 Department of Pathology, Faculty of Medicine, Dalhousie University, Halifax, Canada; 10 Department of Anatomical Pathology, QEII Hospital, Nova Scotia Health, Halifax, Canada

**Keywords:** Oral epithelial dysplasia, Oral cancer, 16S rRNA sequencing, Oral microbiota, Microbiome

## Abstract

A growing body of research associates the oral microbiome and oral cancer. Well-characterized clinical samples with outcome data are required to establish relevant associations between the microbiota and disease. The objective of this study was to characterize the community variations and the functional implications of the microbiome in low-grade oral epithelial dysplasia (OED) using 16S rRNA gene sequencing from annotated archival swabs in progressing (P) and non-progressing (NP) OED. We characterised the microbial community in 90 OED samples — 30 swabs from low-grade OED that progressed to cancer (cases) and 60 swabs from low-grade OED that did not progress after a minimum of 5 years of follow up (matched control subjects). There were small but significant differences between P and NP samples in terms of alpha diversity as well as beta diversity in conjunction with other clinical factors such as age and smoking status for both taxa and functional predictions. Across all samples, the most abundant genus was *Streptococcus*, followed by *Haemophilus*, *Rothia*, and *Neisseria.* Taxa and predicted functions were identified that were significantly differentially abundant with progression status (all Ps and NPs), when samples were grouped broadly by the number of years between sampling and progression or in specific time to progression for Ps only. However, these differentially abundant features were typically present only at low abundances. For example, *Campylobacter* was present in slightly higher abundance in Ps (1.72%) than NPs (1.41%) and this difference was significant when Ps were grouped by time to progression. Furthermore, several of the significantly differentially abundant functions were linked to the *Campylobacteraceae* family in Ps and may justify further investigation. Larger cohort studies to further explore the microbiome as a potential biomarker of risk in OED are warranted.

**Supplementary Information:**

The online version contains supplementary material available at 10.1186/s12903-023-02911-5.

## Background

Oralcancers pose a major public health challenge. In 2020, there were > 350,000 new cases of oral cancer worldwide, with > 175,00 deaths [1]. In North America, there were 35,310 cases and 7,110 deaths, respectively [2]. In Canada, oral cancer represents 3.3% of overall cancer burden in males and 1.5% in females, ranking above stomach, liver, brain, testicular and cervical cancer [3]. The 5-year survival rate for oral cancers remains < 50% in much of the world [1], mainly due to late stage diagnosis [4, 5]. Early detection is vital to the improvement of this prognosis [6]. While the costs to health care systems for oral cancer treatments are large [7], these are decreased with earlier intervention [8].

Oral cancer is frequently preceded by an oral potentially malignant lesion (OPML) [9]. However, even when an OPML is discovered early, clinical management can be challenging, as it is difficult to discriminate between indolent and aggressive disease. A histological diagnosis of oral epithelial dysplasia (OED) provides an indication of risk for high-grade (severe) dysplasia, however, it is a relatively poor prognosticator for low-grade (mild or moderate) dysplasia, which represents the majority of cases [10]. A major barrier to the clinical management of low-grade lesions is the inability to accurately discriminate between indolent and transformative disease [11–15]. Treatment can bear significant morbidity, and given that most low-grade lesions will not progress, it is correspondingly important to avoid overtreatment [16]. Biomarkers are needed to supplement epithelial dysplasia grading in order to triage low-grade OED according to their risk of progression [9, 17].

A growing body of research draws attention to the connection between the microbiome and cancer in the oral cavity. Since *Helicobacter pylori* was first demonstrated to be a causative agent in gastric cancer [18], many efforts have been made to explore the relationship between bacteria and cancer in other body sites. These efforts uncovered additional associations, including *Salmonella typhi* with gallbladder cancer [19], *Bacteroides fragilis* with colon cancer [20], and *Porphyromonas gingivalis* and *Fusobacterium nucleatum *with colorectal cancers [21–23]. In the context of oral cancer, there is growing evidence of the carcinogenicity of bacteria that have been found to inhibit apoptosis, activate cellular proliferation, promote cellular invasion and induce chronic inflammation, although these findings are primarily from in vitro and animal studies [24]. However, given the oral cavity contains more than 700 microbial species that form complex and diverse communities (microbiomes), the microbiome-host interaction is believed to extend far beyond the involvement of a select few species [25]. There has been an increase in the number of studies exploring the microbiome associated with oral squamous cell carcinoma (OSCC) [24, 26–29]. Evidence is growing that polymorphic microbiomes can have an impact on cancer, and have recently been added as an enabling characteristic to the Hallmarks of Cancer [30].

Definitive evidence that the microbiome plays a causal role in the development of oral cancer remains lacking. Shifts in the microbial populations that colonize human tissues have been shown to affect host biological pathways through the output of small molecules and metabolites [31]. Although some research has investigated the diversity of the microbiome in the context of OPML, the studies are cross-sectional or have focused on differences in the oral microbiota associated with health and disease [32, 33]. Longitudinal studies that focus on the relationship between the oral microbiome and the malignant transformation of OED are needed**.**

Well-characterized clinical samples with long follow-up data are required to establish relevant associations between the microbiota and disease. Unlike some anatomical sites, the oral cavity is easily accessible for sampling using non-invasive techniques, for example brush swabs from OPMLs.

The objectives of this study were to characterize the community variations and the functional implications of the microbiome in low-grade OED with known outcome using 16S rRNA gene sequencing from annotated archival brush swabs.

## Methods

### Patient population and study design

This project used data and samples already collected from subjects enrolled in the Oral Cancer Prediction Longitudinal (OCPL) study, an ongoing prospective cohort study being conducted in British Columbia that has recruited over 600 patients with biopsy confirmed OED. Subjects were identified through a centralized population-based biopsy service, the BC Oral Biopsy Service (OBS), where community dentists and surgeons across British Columbia (estimated population 5.2 million, in 2021 [34]) send biopsies for histopathological diagnosis. Patients with a diagnosis of low-grade OED were referred for follow up to OCPL oral dysplasia clinics where they were invited to participate in the OCPL study and were subsequently followed at 6-month intervals, creating an extensive biobank with associated demographic, clinical, histological, and outcome data. Participants were recruited by written informed consent. Details about the OCPL cohort recruitment, sample collection, and participant follow-up procedures have been published previously [13, 35]. As part of the OCPL Study, a cytology brush swab (Innovatek Inc.) was used to collect exfoliated cells from the primary oral lesion by stroking with pressure 10 times in one direction, turning the swab 180° and stroking 10 times in the opposite direction. The brush tip was broken off and placed into a 2 mL vial containing PreservCyt solution preservative (Hologic, Canada). All samples were barcoded, labeled and stored in a secure biobank. Comparative biopsies were performed on the index lesion every 24 months or upon significant clinical change by a certified oral medicine specialist. Histopathological grading was done at the Oral Biopsy Service and was reviewed and confirmed by the study pathologist (LZ), using diagnostic criteria established by the WHO [36].

The present study used a nested, retrospective matched case–control design. Inclusion criteria included being an OCPL study participant with a biopsy confirmed diagnosis of low-grade (mild or moderate) OED [36], and a baseline cytological specimen available. Participants with a previous history of oral cancer were excluded. Demographic, risk-habit, clinical and outcome information of eligible participants were obtained from the study database. Cases consisted of lesions that progressed to severe dysplasia, carcinoma *in-situ *or OSCC (progressors (P)). Controls consisted of lesions that did not progress after a minimum of 5 years of follow-up (non-progressors (NP)). Cases and controls were matched by anatomical site, age (± 6 years), sex, ethnicity, smoking status, and alcohol consumption. A sample size of 83 (28 cases, 55 controls) was required to detect a significant difference with a ratio of two controls to one case, a hypothetical proportion of controls with 20% exposure, and a hypothetical proportion of 50% of cases with exposure, with a significance level of 5% and 80% power on 2-tailed tests in an unmatched case–control study design (OpenEpi® Version 3.01 software) [37]. Thirty cases and 60 controls were pulled to allow for a reasonable margin of error. A simple random sampling method of all eligible participants was employed. Ethics approval for the present study was obtained from the UBC BC Cancer (H20-00,809) and the Dalhousie University Research Ethics Boards (2020–5102). To ensure that the clinical characteristics of the samples would not bias the observations of microbial taxa and associations with progression, a Cox proportional hazards model within the R package survminer [38] was utilized to determine whether time to progression was associated with clinical characteristics: alcohol consumption (drinks per week), smoking history (pack-years), age (years) and grade of dysplasia.

### Sample preparation and 16S rRNA sequencing

Lesion cytology brush swabs from baseline visits were pulled from the OCPL biobank. DNA isolation was performed using the DNeasy Blood & Tissue Kit (QIAGEN, Hilden Germany). An enzymatic lysis buffer preparation and incubation period was followed by the addition of proteinase K, followed by vortex and incubation. Extraction steps were conducted as per the manufacturer’s instructions. DNA was eluted using 50 µl nuclease free distilled water and stored at − 20 °C. Quantification and quality assessment were performed using a spectrophotometer (NanoDrop ND-100; PEQLAB Biotechnologie, Erlangen, Germany).

A 16S rRNA gene sequencing approach was employed to uncover associations between microbial taxa and malignant progression. A single round of PCR (25 cycles) was done using Platinum SuperFi II DNA Polymerase–High-Fidelity PCR Enzyme for preamplification and primers targeting the V1-V3 region (27Fmod forward primer = AGRGTTTGATCMTGGCTCAG; 519R reverse primer = GWATTACCGCGGCKGCTG) [39] of the 16S rRNA gene. PCR products were verified visually by gel electrophoresis. Amplicon fragments were sent to the Integrated Microbiome Resource at Dalhousie University (https://imr.bio) and were PCR-amplified using high-fidelity Phusion Plus® polymerase (New England Biolabs Inc.). Amplified DNA concentrations were then normalized, pooled, and sequenced on an Illumina MiSeq using 300 bp paired end read chemistry.

### Bioinformatic analyses

#### Read processing

Raw forward reads were imported into QIIME2 v2022.2 [40] for processing. Due to low reverse read quality, these were not used. Briefly, primers were trimmed using Cutadapt [41], reads were quality filtered using the default parameters within the quality-filter plugin and reads were denoised using the DADA2 denoising algorithm [42] with 5 errors allowed. Taxonomy was assigned to the resulting Amplicon Sequence Variants (ASVs) using the scikit-learn [43] naïve bayes classifier trained on the full-length 16S rRNA gene SILVA reference database (version 138) [44] downloaded from the QIIME2 website on 4^th^ July 2022. ASVs were removed from further analysis that were: unclassified at the phylum level, classified as mitochondria or chloroplasts, had a maximum abundance of < 10 reads per sample or present in < 3 samples. The resulting ASVs were subsequently classified using a local BLAST [45]search against the SILVA reference database (version 138) [44] and with the scikit-learn [43] naïve bayes classifier trained on the full-length 16S rRNA gene Human Oral Microbiome Database (HOMD; version 15.22) [46]. Unless otherwise stated, the taxonomic classifications used are those obtained from the naïve bayes classifier trained on the full-length 16S rRNA gene HOMD (version 15.22). A phylogenetic tree was built using SEPP [47] with a reference phylogeny created using the SILVA reference database (version 128) [44]. Rarefaction curves were visualised, and all samples were found to have sufficient sampling depth (mean 19,082 reads per sample; range 7,907–48,793). To assess whether shifts in the oral microbiome community were associated with changes in community metabolism, the PICRUSt2 tool was used to predict metabolic pathways as well as Enzyme Commission (EC) numbers [48]. This tool uses 16S rRNA gene sequences and previously published genomic information to estimate the metabolic capabilities of microbial communities. Downstream bioinformatic analyses were performed using R (v4.1.2), Python (v3.9.12) and RStudio (v2022.02.1).

#### Statistical analysis

Raw count tables were normalised by rarefying to the lowest read depth, conversion to relative abundances or conversion to centered log ratios (CLR). The phylogenetic tree was collapsed at different taxonomic ranks using the R package Phyloseq [49]. Alpha diversity was assessed using Chao1 richness, Shannon diversity, Simpson’s diversity, Simpson’s evenness, and Faith’s phylogenetic diversity on rarefied data. Beta diversity between Ps and NPs was assessed using Weighted UniFrac distance [50] on rarefied data as well as the compositionally-aware Phylogenetic Isometric Log Ratio (PhILR) distance [51] on raw count tables and visualized using a principal coordinate analysis (PCoA). Alpha and beta diversity metrics as well as ordinations were calculated using the Python package scikit-bio [52]. Mann–Whitney U tests were run using the Python package scipy [53] to determine whether there were differences in alpha diversity between Ps and NPs. To assess the association between microbial composition and progressor status, PERMANOVA tests were run using the Adonis function within the R package vegan [54] with the following metadata variables: Age, Sex, Ethnicity, Alcohol intake, Smoking status, Grade of dysplasia and Anatomical site. The matched P/NP groupings were given to the model using the strata option to constrain permutations and the tests were run separately with: (i) Progression grouped to Ps/NPs; (ii) Progression grouped to NPs or groupings of the time to progression (< 1, 1–2, 2–3, 3–4, 4–6 or 6 + years); or (iii) the number of months to progression for Ps only. PERMANOVA tests were run separately for Weighted UniFrac and PhILR distances (as calculated above).

To identify taxonomic features, predicted EC numbers or MetaCyc metabolic pathways associated with progression status, we ran MaAsLin2 [55] using the Maaslin2 R package. These tests were run separately with: (i) Progression grouped to Ps/NPs; (ii) Progression groups to NPs or groupings of the time to progression (< 1, 1–2, 2–3, 3–4, 4–6 or 6 + years); or (iii) the number of months to progression for Ps only as fixed effects. The matched P/NP groupings were given to the model as random effects and the NP group was used as the reference. These tests were run with and without the inclusion of the other clinical variables (age, sex, ethnicity [with white used as the reference], alcohol intake, smoking status, grade of dysplasia and lesion site) as fixed effects and with both relative abundance and CLR-transformed data. Taxa, EC numbers or pathways were considered to be significantly differentially abundant between groups if they had a *q*-value of 0.25 (the default in MaAsLin2). JarrVis [56, 57] was used to visualise the links between taxonomy and the top 10 differentially abundant EC numbers. The alpha diversity of ASVs contributing to the top 10 differentially abundant EC numbers were calculated as in the FuncDiv R package (https://github.com/gavinmdouglas/FuncDiv) [58].

Alpha and beta diversity analyses as well as PERMANOVA and differential abundance tests were run in the same way for taxonomic data at the ASV, species or genus level as well as on the PICRUSt2 output at the predicted pathway or enzyme level. All code used for analysis can be found at https://github.com/R-Wright-1/OED_microbiome and https://doi.org/10.5281/zenodo.7093667. Sequences were deposited at Gene Expression Omnibus (GEO) with accession GSE198811.

## Results

### Demographic analysis of the cohort

**Fig. 1:**
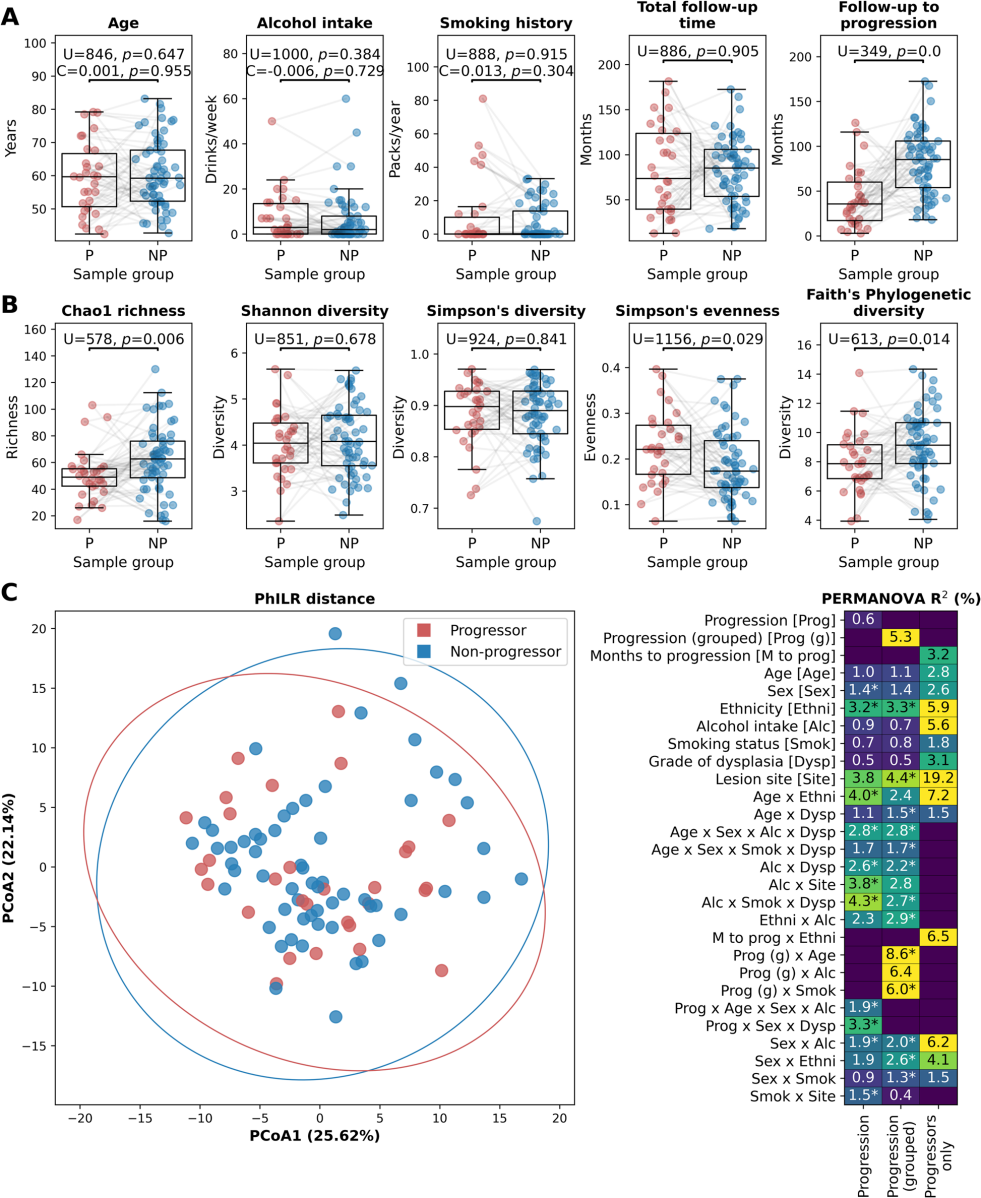
Clinical summary and sample diversity at the species level. **A** Age, alcohol intake, smoking status, and follow-up time were similar between groups. **B** Species diversity within the microbial communities of 30 progressing (P) and 60 non-progressing (NP) oral epithelial dysplasia (OED) samples using five different alpha-diversity metrics: Chao1 richness, Shannon diversity, Simpson’s diversity, Simpson’s evenness, and Faith’s phylogenetic diversity. U and *p*-values in **A** and **B** were determined by Mann–Whitney U tests and lines indicate matched Ps and NPs. For age, alcohol intake and smoking history, Cox proportional hazard test coefficients (C) and *p*-values are shown in addition to the Mann–Whitney U test statistics. Lines between points indicate matched Ps/NPs and boxes show the median, upper and lower quartiles while whiskers show the range of the data (1.5 times the interquartile range). **C** Principal Coordinates Analysis (PCoA) and PERMANOVA tests using Phylogenetic Isometric Log Ratio (PhILR) distance at the species level (Supp. Table S1C). Ellipses show the confidence interval (3 standard deviations) for each group and the values shown on each axis label indicate the proportion of sample variation accounted for by that axis. The heatmap in **C** shows PERMANOVA *R*^2^ values for all separate variables (shown with abbreviations in the first instance) that were added to the models as well as all interactions between variables with an *R*^2^ ≥ 5% and/or with *p* ≤ 0.05 (denoted with an asterisk). All PERMANOVA *R*^2^ and *p* values are shown in Supp. Table S1C. The columns show PERMANOVA tests for Progression (P/NP), Progression (grouped; NP and P grouped to < 1, 1–2, 2–3, 3–4, 4–6 or 6 + years for time to progression) and Progressors only with the specific follow-up time. The equivalent of **C** for Weighted UniFrac distance is shown in Supp. Fig. S3

Participants were followed to an average of 83.8 months (Table 1; Supp. Table S1A). The average age at diagnosis was 60.2 years and the ratio of males (*n* = 42) to females (*n* = 53) was almost equal. Participants identified as primarily white (81%), followed by Asian (11%), and South or East Asian (6%). The majority of participants reported having never smoked (60%) and were non- or light alcohol drinkers (90%). Ninety samples were included in the study: 30 cases (progressors; OED that progressed to severe dysplasia, *CIS* or SCC) and 60 controls (non-progressors; OED that did not progress after a minimum of 5 years of follow-up). Samples were primarily from the lateral or ventral tongue or from the floor of mouth. Progressors (Ps) and non-progressors (NPs) were matched by clinical and demographic variables, and there were therefore no significant differences in lesion site, age, sex, smoking history or alcohol consumption between Ps and NPs (*p* > 0.05). Cohorts were followed for a comparable amount of time. Given that the oral cavity may be exposed to a variety of environmental carcinogens, and lifestyle is a large factor contributing to this, we assessed the effects of various clinical characteristics on the progression status of our cohort. The age, smoking status, and alcohol intake did not differ significantly between P and NP sample sets (Mann–Whitney U tests *p* > 0.384) and were not associated with disease onset time (time to progression; Cox proportional hazards test *p* > 0.3; Fig. 1A), and the cohorts were followed for a comparable amount of time (Table 1). 53% (*n* = 48) of samples exhibited moderate OED and 47% (*n* = 42) demonstrated mild OED. As expected, grade of dysplasia was significant for risk of progression (coefficient 0.96, *p* = 0.0194), with a greater proportion of Ps exhibiting moderate dysplasia (70%). Therefore, to control for potential confounding, multivariable analyses were performed.

**Table 1:** Clinicopathological information of patient cohort

	All (%)^£^	No progression^*^ (%)^£^	Progression^*^ (%)^£^
**Total**	*n* = 90	*n* = 60	*n* = 30
Length of follow-up Median months (range)	83.8 (12.9 to 181.4)	85.3 (18.0 to 172.5)	73.7 (12.9 ± 181.4)
Age at diagnosis Mean (years ± SD)	60.2 ± 10.4	60.6 ± 10.2	59.3 ± 10.9
**Sex**			
Male	42 (47)	28 (47)	14 (47)
Female	48 (53)	32 (53)	16 (53)
**Ethnicity**			
White	73 (81)	49 (82)	24 (80)
Asian	10 (11)	6 (10)	4 (13)
South or East Asian	5 (6)	5 (8)	0 (0)
Other	2 (2)	0 (0)	2 (7)
**Smoking history**^a^			
Never	54 (60)	35 (58)	19 (63)
Ever	36 (40)	25 (42)	11 (37)
**Alcohol consumption**^b^			
Non/light	81 (90)	54 (90)	27 (90)
Heavy	9 (10)	6 (10)	3 (10)
**Lesion site**^c^			
Low Risk	12 (13)	8 (13)	4 (13)
High Risk	78 (87)	52 (87)	26 (87)
**Grade of dysplasia**			
Mild dysplasia	42 (47)	33 (55)	9 (30)
Moderate dysplasia	48 (53)	27 (45)	21 (70)
**Time to progression category**			
< 1 year	-	-	5 (17)
1 – 2 years			5 (17)
2 – 3 years			5 (17)
3 – 4 years			6 (20)
4 – 6 years			5 (17)
> 6 years			4 (13)

^*^Progression = progression to severe dysplasia, carcinoma in situ, or squamous cell carcinoma; No progression = no progression to severe dysplasia, carcinoma in situ, or squamous cell carcinoma after a minimum of five years of follow-up

^£^Column percentage reported

^a^Never smoker < 100 cigarettes in lifetime; Ever smoker > 100 cigarettes in lifetime

^b^Heavy alcohol consumption is defined as consumption of more than 14 drinks per week for females and 21 drinks per week for men. One alcoholic drink was defined as 8 oz of beer, 5 oz of wine or 1 oz of spirits

^c^High Risk = floor of mouth, soft palate, and tongue; Low Risk = all other sites

### Establishing a profile of microbial taxa in progressing and non-progressing OED

#### Diversity of oral microbial communities

Since external clinical factors did not have a significant impact on disease onset, we investigated whether changes in the microenvironment may be allowing for the colonization of additional organisms not previously known to be associated with the oral cavity which may impact the progression of OED. ASVs were classified taxonomically using the HOMD version 15.22 [46] (see Supp. Results and Supp. Figs. S1 and S2 for the results obtained using different taxonomic classifiers/databases).

To assess the diversity at the species level within the microbial communities of P and NP OED samples (Supp. Table S1B), we used five different alpha-diversity metrics (Fig. 1B). Although Ps and NPs showed similar levels of species diversity according to Shannon and Simpson’s diversity metrics, there was a relative decrease in diversity according to Chao1 richness and Faith’s PD (Mann–Whitney U test, *p* = 0.006 and *p* = 0.014, respectively) and an increase in Simpson’s evenness (Mann–Whitney U test *p* = 0.029) for the Ps relative to NPs. This may indicate that while there is a reasonably equal abundance of species within each sample set, the number of unique species may be lower in Ps, and they may be more closely related in phylogenetic space. Therefore, we observe lower relative diversity of microbes in the oral cavity of Ps.

We compared the microbial diversity of Ps and NPs using both PhILR (Fig. 1C) and Weighted UniFrac distance (Supp. Fig. S3) with PERMANOVA tests with age, sex, ethnicity, alcohol intake, smoking status, grade of dysplasia, lesion site and progression grouped in one of three ways: (i) Ps and NPs (Progression; residual *R*^2^ = 6.1% and 0.056, respectively); (ii) Ps grouped by the number of years to progression (< 1, 1–2, 2–3, 3–4, 4–6 and 6 + ; Progression (Grouped); Residual *R*^2^ = 5.2% and 0.050, respectively) and NPs; (iii) Ps only with the number of months to progression (Progressors only; Residual *R*^2^ = 0 for both). Information on the matching of Ps and NPs was also given to the first two models. While there was no statistical difference observed in any of these groupings (PERMANOVA *p* > 0.05; Fig. 1C and Supp. Fig. S3) and the Principal Coordinates Analysis (PCoA) of PhILR distance showed no clustering of P and NP samples – indicating that diversity was not affected by progression status alone – there were some significant (*p* ≤ 0.05) differences with some other clinical variables (sex, ethnicity and smoking status) and interactions between progression and the other clinical variables (Fig. 1C and Supp. Fig. S3). Of note, for PhILR distance there were significant interactions between (i) progression and age, sex and alcohol intake (*R*^2^ = 1.9%, *p* = 0.027) or sex and grade of dysplasia (*R*^2^ = 3.3%, *p* = 0.017) (ii) progression (grouped) and age (*R*^2^ = 8.6%, *p* = 0.025) or smoking status (*R*^2^ = 6%, *p* = 0.004). The results were similar for Weighted UniFrac distance (Supp. Fig. S3), with diversity not being significantly affected by progression status alone, with significant interactions between (i) progression and sex (*R*^2^ = 2.4%, *p* = 0.011), age, sex and alcohol intake (*R*^2^ = 1.5%, *p* = 0.043) or sex and ethnicity (*R*^2^ = 0.7%, *p* = 0.049) and (ii) progression (grouped) and either age (*R*^2^ = 8.2%, *p* = 0.036) or smoking status (*R*^2^ = 6.3%, *p* = 0.001). While there were no significant differences found with any of the variables for (iii) Ps only, the lesion site was contributing to large (non-significant) differences in beta diversity (*R*^2^ = 19.2% or 20.5% for PhILR or Weighted UniFrac distance, respectively).

#### Phylum- and genus-level shifts in microbial communities

**Fig. 2:**
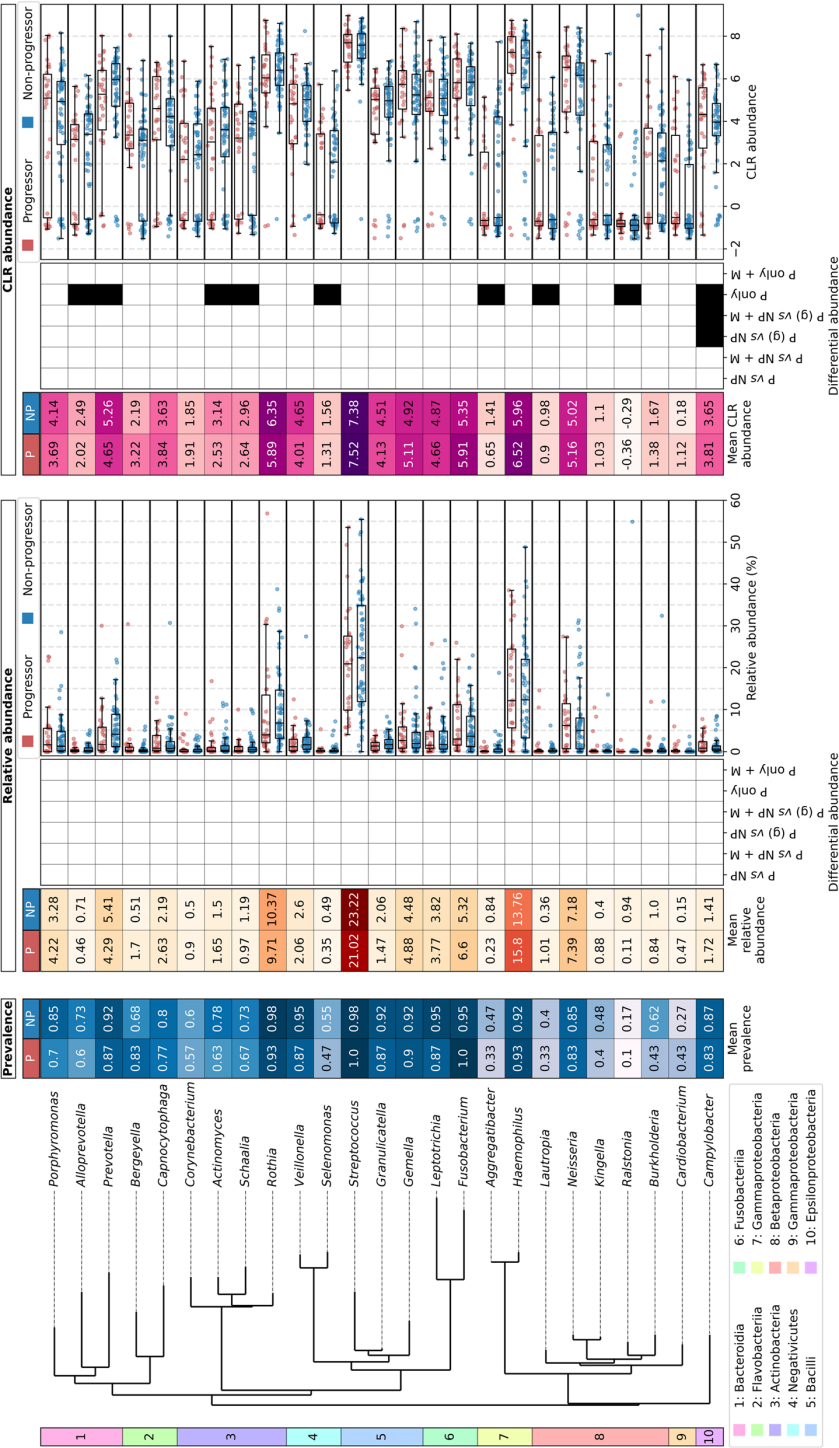
Prevalence, abundance and differential abundance of the top 25 most abundant genera in progressor and non-progressor samples. Phylogenetic tree showing the class of each genus and a heatmap showing mean prevalence (blue scale; left). Heatmaps showing mean abundance of genera in progressor (P) or non-progressor (NP) samples (left) or differential abundance (centre) are shown alongside boxplots showing abundance in all samples (right) for each of relative abundance and CLR abundance. In the boxplots, each sample is shown as an individual point and boxes show the median, upper and lower quartiles while whiskers show the range of the data (1.5 times the interquartile range). MaAsLin2 tests for differential abundance were run with (+ M) and without the other clinical variables (metadata, M; age, sex, ethnicity, alcohol intake, smoking status, grade of dysplasia and lesion site). As above for PERMANOVA tests, progression was grouped in one of three ways: (i) Ps and NPs (P *vs* NP); (ii) Ps grouped by the number of years to progression (< 1, 1–2, 2–3, 3–4, 4–6 and 6 + ; P *vs* NP); and (iii) Ps only with the number of months to progression (P only). For (i) and (ii) the matched P/NP grouping was given to the model so only matched controls were used. Genera were determined to be differentially abundant and are shown in black in the heatmap if they had *q* ≤ 0.25 (the default in MaAsLin2). White denotes that they were not significantly differentially abundant

When taxa in Ps and NPs were compared, small differences in abundance were observable up to the phylum level of classification (Supp. Fig. S4). We have examined the abundance of taxa using both relative abundance, as this is what the majority of studies to date have used, and CLR abundance, as this accounts for the compositionality of microbiome data [59]. For the relative abundances, higher values indicate that more sequences belonging to a particular taxon are present. For the CLR abundance, a zero value indicates that the abundance of a taxon is equal to the mean log_2_ abundance of all taxa, with positive or negative values indicating higher or lower abundances than the mean log_2_ relative abundance, respectively. In both sample groups, the dominant phyla were *Firmicutes*, *Proteobacteria*, *Actinobacteria*, *Bacteroidetes*, and *Fusobacteria*; a slight decrease in *Firmicutes* and a slight increase in *Proteobacteria* in Ps was observed for both relative and CLR abundance (Supp. Fig. S4A). At the genus-level, *Streptococcus* was the most abundant, with *Haemophilus*, *Neisseria*, and *Rothia* also making up high abundances for both relative and CLR abudance (Fig. 2; Supp. Fig. S4E). The relative abundances of these genera between NPs and Ps appear quite similar, other than an apparent decrease in *Streptococcus* in Ps.

#### Known oral cavity colonizers did not correlate with progression status

Most of the more abundant taxa identified in this study are known to be associated with human oral health [60, 61]. However, *Streptococcus* spp., *Haemophilus*, and *Fusobacterium*have been correlated with oral cancer and epithelial precursor lesions [26, 27, 33, 60, 62, 63]. Furthermore, some of the less abundant taxa identified, including *Campylobacter, Prevotella, Pseudomonas,* and *Rothia*, have been shown to be differentially enriched in studies that have investigated OSCC or OPML by swab or biopsy [26, 27, 33, 64]. We therefore carried out differential abundance testing between Ps and NPs using MaAsLin2 [55] – which allows the inclusion of the matched P/NP grouping as well as other metadata – at the genus and species levels in order to see whether there was any indication that these taxa are significantly associated (*q* ≤ 0.25, the default cut-off in MaAsLin2) with progression status. We ran these tests both with and without the other clinical variables (age, sex, ethnicity, alcohol intake, smoking status, grade of dysplasia and lesion site) as well as with relative abundance or CLR-transformed count tables. There were no genera that were significantly differentially abundant with progression status or time to progression with the relative abundance data, however, there were some with the CLR abundance data. For the CLR abundance, no genera were significantly differentially abundant between Ps and NPs, however, when Ps were grouped to the time to progression (< 1, 1–2, 2–3, 3–4, 4–6 or 6 + years), one genus was significant in the CLR-transformed data, without or with the inclusion of the other clinical variables: *Campylobacter*, which was typically more abundant in Ps than NPs, although the magnitude and direction of this difference varied depending on the time to progression (Fig. 2, Supp. Fig. S5 and Supp. Table S2). There were 124 genera that were significantly differentially abundant with follow-up time in the Ps only (without the inclusion of the other clinical variables), nine of which were among the top 25 most abundant genera; *Actinomyces*, *Aggregatibacter*, *Alloprevotella*, *Campylobacter*, *Lautropia*, *Prevotella*, *Ralstonia*, *Schaalia* and *Selenomonas*. However, none of these were significantly differentially abundant when the other clinical variables were also included (Fig. 2 and Supp. Table S2).

**Fig. 3:**
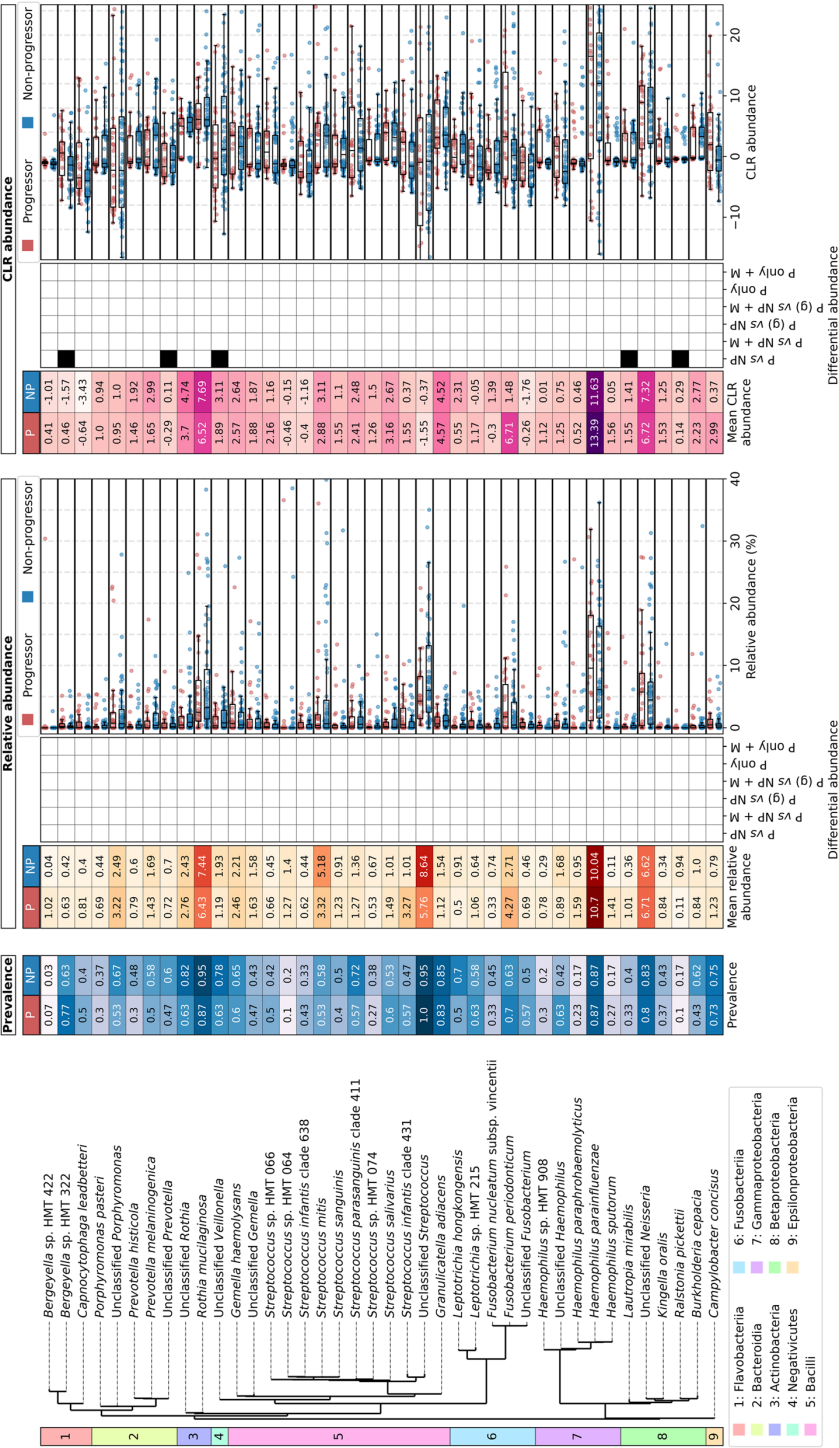
Prevalence, abundance and differential abundance of the top 40 most abundant species in progressor and non-progressor samples. Phylogenetic tree showing the class of each species and a heatmap showing mean prevalence (blue scale; left). Heatmaps showing mean abundance of species in progressor (P) or non-progressor (NP) samples (left) or differential abundance (centre) are shown alongside boxplots showing abundance in all samples (right) for each of relative abundance and CLR abundance. In the boxplots, each sample is shown as an individual point and boxes show the median, upper and lower quartiles while whiskers show the range of the data (1.5 times the interquartile range). MaAsLin2 tests for differential abundance were run with (+ M) and without the other clinical variables (metadata, M; age, sex, ethnicity, alcohol intake, smoking status, grade of dysplasia and lesion site). As above for PERMANOVA tests, progression was grouped in one of three ways: (i) Ps and NPs (P *vs* NP); (ii) Ps grouped by the number of years to progression (< 1, 1–2, 2–3, 3–4, 4–6 and 6 + ; P *vs* NP); and (iii) Ps only with the number of months to progression (P only). For (i) and (ii) the matched P/NP grouping was given to the model so only matched controls were used. Species were determined to be differentially abundant and are shown in black in the heatmap if they had *q* ≤ 0.25 (the default in MaAsLin2). White denotes that they were not significantly differentially abundant

At the species level there were – as for the genus level – no taxa that were significantly differentially abundant with either progression status or time to progression with the relative abundance data, but there were with the CLR abundance. There were 271 species that were significantly differentially abundant between Ps and NPs (five when the other clinical variables were also included) the majority of which were present in only very low abundances (Supp. Table S3). Of these 271 species, only five were present within the top 40 most abundant species (Fig. 3 and Supp. Table S3): two of these were higher in abundance in Ps than NPs (*Bergeyella* sp. HMT 322 and *Lautropia mirabilis*), two were lower in abundance in Ps than NPs (unclassified *Veillonella* and *Ralstonia pickettii*) and one was very similar in abundance between Ps and NPs, with slightly higher CLR abundance in NPs and higher relative abundance in Ps (unclassified *Prevotella*). None of these were also significantly differentially abundant with the inclusion of the clinical variables. Three species were significantly differentially abundant when Ps were grouped to the time to progression (< 1, 1–2, 2–3, 3–4, 4–6 or 6 + years), two of which were detected without (*Gemella morbillorum* and *Neisseria elongata*) and one with the inclusion of the other clinical variables (*Prevotella pallens*), although none of these were within the 40 most abundant species (Fig. 3 and Supp. Table S3). No species were significantly differentially abundant with time to progression for Ps only.

#### Metabolic impact of oral microbiome changes associated with progression

Colonizing bacteria are known to interact with their hosts through both direct and indirect mechanisms, including receptor interactions and the release of nutrients or metabolic products into the microenvironment. Therefore, we next examined the involvement of specific EC numbers or metabolic pathways in NPs and Ps using PICRUSt2 to generate predicted metagenomes for all samples based on the taxa present (Supp. Tables S1D and S1E). The alpha and beta diversity of profiles generated by PICRUSt2 for functions at the level of both genes for EC numbers and MetaCyc metabolic pathways were similar to that of the taxa (Fig. 1 and Supp. Figs. S6 and S7). Ps have significantly lower Chao1 richness and significantly higher Simpson’s evenness than NPs (Mann–Whitney U test *p* < 0.05) and while progression status did not have a significant effect on Bray–Curtis beta diversity on its own, it did have a significant interaction with several of the other clinical variables (*e.g.*, sex, smoking status, lesion site and grade of dysplasia; Supp. Figs. S6 and S7 and Table S1C).

We carried out differential abundance testing using MaAsLin2 [55] at both the enzyme and the pathway level, with both relative and CLR abundance and both with and without the other clinical variables (age, sex, ethnicity, alcohol intake, smoking status, grade of dysplasia and lesion site). We initially examined the pathway level, and, as with the taxa, there were no significant differences between Ps and NPs for the relative abundance data, but there were five pathways that were significantly differentially abundant between Ps and NPs for the CLR abundance data without the other clinical variables, with one of these pathways (PWY0-1533; generally higher in abundance in NPs than Ps) still being differentially abundant with the inclusion of the other clinical variables (Supp. Table S4 and Supp. Fig. S8). When Ps were grouped to the time to progression (< 1, 1–2, 2–3, 3–4, 4–6 or 6 + years), there were 21 pathways that were significantly differentially abundant between one or more groups and the NPs without the other clinical variables, six of which were also significant with the inclusion of the other clinical variables (P164-PWY, P562-PWY, PWY-5265, PWY-6608 and RHAMCAT-PWY; along with one additional pathway; Supp. Fig. S8). For progressors only, there was only one pathway that was significantly differentially abundant (for the CLR abundance with the other clinical variables); P221-PWY (Supp. Table S4 and Supp. Fig. S8).

**Fig. 4:**
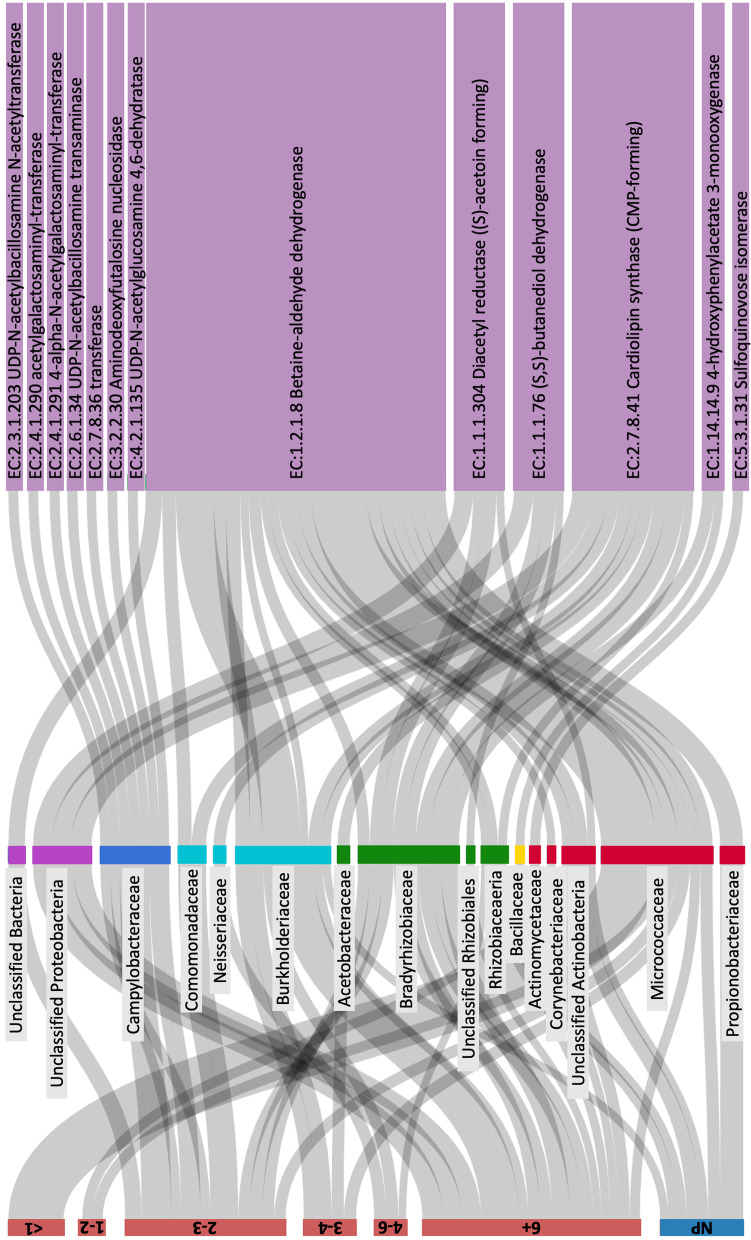
JarrVis sankey plot showing the links between samples grouped by years to progression (left), microbial families (middle) and predicted EC numbers (right). Microbial families from different classes have differently coloured nodes. The sizes of nodes and connecting lines correspond to mean abundance values (rarefied gene copy numbers) for the contribution to microbial families and predicted EC numbers within each sample grouping. Only families/EC numbers with a mean abundance of > 100 copies are shown

When we examined the enzymes, there were again very few that were significantly differentially abundant with progression status for the relative abundance, but there were a large amount that were for the CLR abundance (Supp. Table S5). We therefore focus only on the enzymes that were identified both with and without the inclusion of the other clinical variables: six for Ps *vs* NPs and nine for Ps grouped to the time to progression *vs* NPs (Supp. Fig. S9; no enzymes were significantly differentially abundant with time to progression for progressors only). In order to explore the links between taxonomy and function, we examined the ASVs that were contributing to these 15 EC numbers and found that the alpha diversity tended to be higher as the abundance of the EC number increased (Supp. Fig. S10). We used JarrVis to collapse the ASVs contributing to these EC numbers at the family level and visualise those families/EC numbers with > 100 gene copies on average within a sample grouping (after rarefying; Fig. 4). Two of the EC numbers were removed by this filtering due to low abundance. This revealed that some families were the only abundant contributors to some enzymes and that these contributions could come from only a single sample group, *i.e.*, the contribution of Campylobacteraceae to enzymes EC:2.3.1.203, EC:2.4.1.290, EC:2.4.1.291, EC:2.6.1.34, EC:2.7.8.36, EC:3.2.2.30 and EC:4.2.1.135 from the 2–3 year time to progression group. There were also a few families for which there were only abundant contributions from one sample grouping (*e.g.*, Propionibacteriaceae, NP; Corynebacteriaceae, Bacillaceae, unclassified Proteobacteria and unclassified Rhizobiales, 6 + years; Acetobacteraceae, 3–4 years; Actinomycetaceae, 1–2 years; unclassified Bacteria, 2–3 years; Neisseriaceae, 4–6 years and Comomonadaceae, 2–3 years), and some that only had abundant contributions from either NPs or those groups with a longer time to progression (*e.g.*, Burkholderiaceae, unclassified Actinobacteria and Rhizobiaceae). This also shows that in those that progressed soon after sampling (*i.e.,* the < 1 year group), there was only one family (Micrococcaceae) abundant enough to be visualised, and this family only contributed to one EC number (EC:1.2.1.8 Betaine-aldehyde dehydrogenase).

## Discussion

This is the first study to use archival lesion swabs for the characterization of bacteria within OED. It is also the first to compare the diversity between progressing and non-progressing OED in a longitudinal design and to report on the functional potential of the bacteriome associated with risk of progression in OED. Our study has revealed that the bacterial make-up of the OED niche is similar to what has previously been described in the normal oral cavity, OPML and OSCC at the phylum and genus levels [29, 60, 65–67]. We also found that although participant factors such as age or smoking status have an overall larger impact on the oral microbiome than progression status, once these are controlled for there are also small differences in the microbiome that can be attributed to progression status (Fig. 1). In cross-sectional studies, differential relative abundance of certain genera, such as *Streptococcus*, *Prevotella, Campylobacter, Pseudomonas*, and *Fusobacterium,*is often seen between normal and diseased states [26, 27, 33, 64]. While there were slight differences in relative abundances of these genera, only *Campylobacter* showed significant differences in CLR-transformed abundance while controlling for clinical variables. *Campylobacter*has indeed been shown to be associated with OSCC tissue and is often higher in abundance in tumour tissue than in normal tissue [27, 68–72]. Furthermore, *Campylobacter spp*. has shown increased abundance in oral leukoplakia compared to contra-lateral controls [33]. However, taxonomic features at the species level that were determined to be significantly associated with either Ps or NPs were low abundance, and therefore are not likely to be large contributors to the niche. To determine whether these slight changes in abundances are significant at either genera or species level resolutions, it will likely take substantially larger sample sizes.

More recently, the literature has pointed to the role of the microbial metabolome and how the sum of the community as a whole may play a larger role in influencing the tissue microenvironment than any species alone [73, 74]. The concept of functional redundancy may explain how compositional variations of the microbiome associated with OPML and OSCC may collectively be contributing to a dysbiotic community. Our functional prediction analysis identified 15 enzymes (EC numbers) that were significantly differentially abundant with the time to progression in Ps (Supp. Figs. S9 and S10). In particular, seven of the identified enzymes were linked to the abundance of the *Campylobacteraceae* family in 2–3 year Ps. Several of these (EC:2.4.1.290, EC:2.4.1.291, EC:2.6.1.34) are related to the N-linked glycosylation, which was first described for the bacterial species *Campylobacter jejuni,* which belongs to this family [75], as well as protein glycosylation in general (EC:2.3.1.203). In particular, *N,N*– bacillosamine, a substrate and intermediate of these processes, may contribute to the pathogenicity of the bacteria and play a role as a virulence factor [76]. Amino sugar and nucleotide sugar metabolism were also associated with multiple enzymes (EC:2.3.1.203, EC:2.6.1.34, EC:4.2.1.135) and nucleotide sugar metabolism may provide glycosyl donors for glycosylation [77, 78]. While the specifc mechanisms by which glycoproteins affect pathogencity and virulence are not well-known, they may either be expressed on the surface of bacteria, where they are important for adhesion to host cells and thus the initiation of infection, or secreted by the bacteria, which may allow for the evasion of the host’s immune system [79]. The repeated appearance of enzymes involved in glycosylation may indicate that those bacteria which are inhabiting the oral cavity in pre-malignant lesions that progress behave more pathogenically. However, it is uncertain as to which *Campylobacter* species are particularly abundant and whether they are pathogenic or commensal in more aggressive disease.

Given that our study design compared samples taken at an early stage of disease (mild /moderate OED), the lack of significant differences between P and NPs may indicate that changes in overall diversity as well as taxonomic shifts occur at later stages in progression, or perhaps detectable only after OSCC has been established. Further to this, a study looking at OSCC, normal, and OPML found that while OSCC samples clustered based on beta-diversity, pre-cancer and normal samples were mixed, indicating that there was not a great difference in the diversity between these groups [65]. This may support the notion that microbial changes in diversity change at a later stage. Larger studies that investigate the full spectrum of expertly graded OED are necessary.

A potential limitation of this work is that the samples were stored at ultra-low temperatures for a considerable period of time from date of collection to DNA extraction (mean 15.7 years, range 8.9 – 24.3 years). It is unknown how long-term storage may have affected such small samples with a potentially small biomass. However, this should also be viewed as strength, as older samples have longer follow-up and as a result, more robust outcome data. Alpha-diversity differed between Ps and NPs; however, beta diversity did not differ significantly among these groups alone (although it did in conjunction with other participant metadata). One of the reasons for this lack of significance in beta diversity may be due to a relatively small sample size. It is possible that the study suffered from a type II error due to lack of statistical power. However, prior to moving forward with a full-scale study, a pilot study of these small and invaluable samples was necessary. A significant strength is that this study examined patients with known outcomes, who had samples taken prior to developing disease. However, a limitation is that no longitudinal sampling points have been evaluated. A comparison between longitudinal samples can provide insightful results on the temporal changes. Future studies that employ repeated sampling are warranted. A caveat to this work is that 16S rRNA sequencing is not always capable of providing a high enough resolution to differentiate between closely related genera. In addition, functional profiles were established via prediction analysis (PICRUSt2) [48]. A metagenome sequencing approach may yield more comprehensive data for taxonomic assignment to the species level and provide more direct information on metabolic pathways for functional profiling based on pathway component genes.

## Conclusions

In conclusion, for the first time, we have characterized the microbiome of low-grade OED with known outcome using 16S rRNA gene sequencing from annotated archival swabs. At the genus level, known oral cavity colonizers did not correlate with progression. The collective metabolic impact of the bacteriome trends toward a depletion of several enzymes that have been previously linked to cancer in progressing oral lesions but requires a larger sample size to show this more clearly. Having shown that quality NGS data can be obtained from archival oral swabs, larger prospective cohort studies to further explore the taxa and the function of the microbiome as a potential biomarker of risk in OED are warranted.

## Supplementary Information

**Additional file 1.** Supplementary results text and figures.

**Additional file 2.** Supplementary tables 1A-E showing (**A**) Participant and sample metadata, (**B**) Output from QIIME2 showing amplicon sequence variant (ASV), ASV name, assigned taxonomy, DNA sequence and abundance in samples, (**C**) PERMANOVA results, (**D**) Output from PICRUSt2 showing EC number, description of the enzyme produced and abundance in samples, and (**E**) Output from PICRUSt2 showing pathways, a description of the pathway and abundance in samples.

**Additional file 3.** Supplementary tables 2A-L showing MaAsLin2 differential abundance test results at the genus level.

**Additional file 4.** Supplementary tables 3A-L showing MaAsLin2 differential abundance test results at the species level.

**Additional file 5.** Supplementary tables 4A-L showing MaAsLin2 differential abundance test results on PICRUSt2 predicted metagenomes at the pathway level.

**Additional file 6.** Supplementary tables 5A-L showing MaAsLin2 differential abundance test results on PICRUSt2 predicted metagenomes at the enzyme commission number level.

## Data Availability

Data is publicly available at the Gene Expression Omnibus (GEO) with accession GSE198811.
