# Correction: Exploring the microbiome of oral epithelial dysplasia as a predictor of malignant progression

**DOI:** 10.1186/s12903-024-04773-x

**Published:** 2024-09-04

**Authors:** Robyn J. Wright, Michelle E. Pewarchuk, Erin A. Marshall, Benjamin Murray, Miriam P. Rosin, Denise M. Laronde, Lewei Zhang, Wan L. Lam, Morgan G. I. Langille, Leigha D. Rock

**Affiliations:** 1 Department of Pharmacology, Dalhousie University, Halifax, Canada; 2 Department of Integrative Oncology, British Columbia Cancer Research Centre, Vancouver, Canada; 3 Department of Cancer Control Research, British Columbia Cancer Research Centre, Vancouver, Canada; 4 Department of Biomedical Physiology and Kinesiology, Simon Fraser University, Burnaby, Canada; 5 Faculty of Dentistry, University of British Columbia, Vancouver, Canada; 6 Oral Biopsy Service, Vancouver General Hospital, Vancouver, Canada; 7 Beatrice Hunter Cancer Research Institute, Halifax, Canada; 8 Faculty of Dentistry, Dalhousie University, Halifax, Canada; 9 Department of Pathology, Faculty of Medicine, Dalhousie University, Halifax, Canada; 10 Department of Anatomical Pathology, QEII Hospital, Nova Scotia Health, Halifax, Canada

**Correction to: BMC Oral Health (2023) 23:206**

10.1186/s12903-023-02911-5

In this article [1], the author name “Benjamin Murray” was incorrectly written as “Benjamin Murrary”. The author name is corrected in this version.

And the word “Oralcancers” was changed to “Oral cancers” in the first sentence of the *Background* section.
