# Supplementary material for: Exploring the microbiome of oral epithelial dysplasia as a predictor of malignant progression
Source: BMC Oral Health. 2023 Apr 6;23:206. doi: 10.1186/s12903-023-02911-5 (PMC10080811; doi:10.1186/s12903-023-02911-5)
Supplement: Supplementary file 1 — Additional file 1. Supplementary results text and figures. [file 12903_2023_2911_MOESM1_ESM.docx]

Supplementary Results and Figures for Exploring the microbiome of oral epithelial dysplasia as a predictor of malignant progression

**Robyn J. Wright^1*^; Michelle E. Pewarchuk^2^; Erin A. Marshall^2^; Benjamin Murrary^1^, Miriam P. Rosin^3,4^; Denise M. Laronde^3.5^; Lewei Zhang^5,6^; Wan L. Lam^2^; Morgan G.I. Langille^1,7^; Leigha D. Rock^1,7,8,9,10^**

^1^Department of Pharmacology, Dalhousie University, Halifax, Canada. ^2^Department of Integrative Oncology, British Columbia Cancer Research Centre, Vancouver, Canada. ^3^Department of Cancer Control Research, British Columbia Cancer Research Centre, Vancouver, Canada. ^4^Department of Biomedical Physiology and Kinesiology, Simon Fraser University, Burnaby, Canada. ^5^Faculty of Dentistry, University of British Columbia, Vancouver, Canada. ^6^Oral Biopsy Service, Vancouver General Hospital, Vancouver, Canada ^7^Beatrice Hunter Cancer Research Institute, Halifax, Canada. ^8^Faculty of Dentistry, Dalhousie University, Halifax, Canada.  ^9^Department of Pathology, Faculty of Medicine, Dalhousie University, Halifax, Canada. ^10^Department of Anatomical Pathology, QEII Hospital, Nova Scotia Health, Halifax, Canada.

*** Correspondence:**Robyn Wright, PhD
[Robyn.Wright@dal.ca](mailto:Robyn.Wright@dal.ca)

**Supplementary Results**

To examine the differences between Ps and NPs at different taxonomic ranks, we initially investigated the taxonomic classifications given by three methods: (i) the naïve bayes classifier within QIIME2 with the SILVA reference database (version 138)^1^; (ii) local BLAST searches with the SILVA reference database (version 138) and a simplistic taxa classifier; and (iii) the naïve bayes classifier within QIIME2 with the HOMD (version 15.22)^2^ (**Supp. Table S1B**). As also found by Bokulich *et al.*^3^ for the V1-V3 region of the 16S rRNA gene, the naïve bayes classifier methods perform much better than the BLAST method, method (ii), which was only able to give species-level classifications for 20 of the 2643 ASVs. With method (ii), each ASV had a mean of 26.8 ± 32.4 (median 9.5) matches that were above 97% identity, and with the low number of ASVs with a species-level classification, we did not compare this method further. Methods (i) and (iii) both classified many more ASVs than method (ii), with 1140 or 1977, respectively, of the 2643 ASVs having species-level classifications. We examined these species-level classifications further, and found that the method (iii), with the HOMD, accounted for slightly more variation (PERMANOVA residual *R^2^* = 0.061 for method (iii) and *R^2^* = 0.064 for method (i); **Supp. Table S1C**) and that this was slightly better represented on the first two axes of a PCoA plot (total variation 47.76% for method (iii) and 40.25% for method (i); **Supp. Figure S1**) than method (i) (**Supp. Figure S2**). We therefore use the taxonomic classifications obtained with the HOMD, method (iii), throughout the remainder of the manuscript.

**Supplementary Figures**

| 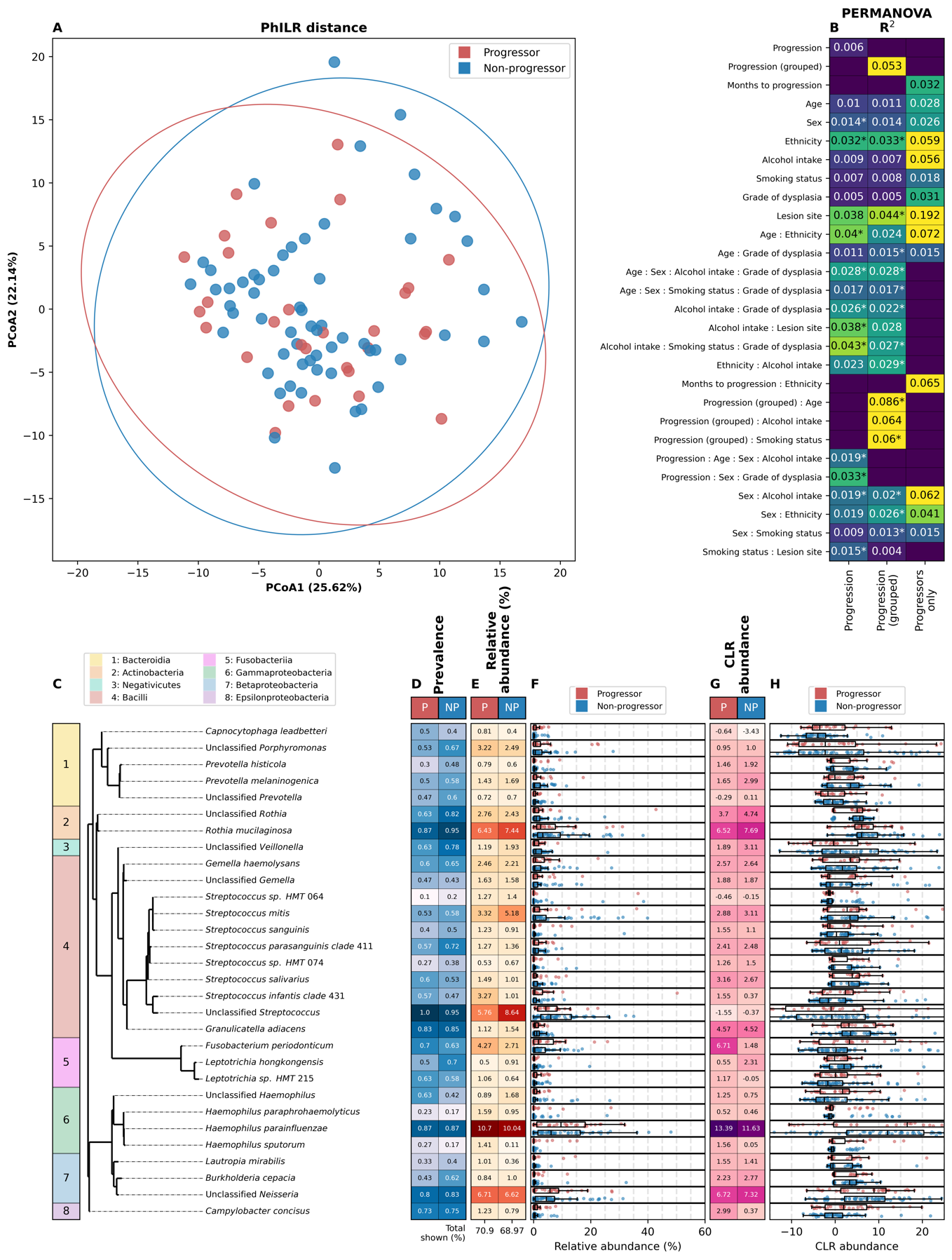  **Supp. Figure 1.** Sample diversity at the species level with taxonomic classifications from the HOMD. (**A**) Principal Coordinates Analysis (PCoA) and (B) PERMANOVA tests using Phylogenetic Isometric Log Ratio (PhILR) distance. Ellipses show the confidence interval (3 standard deviations) for each group and the values shown on each axis label indicate the proportion of sample variation accounted for by that axis. The heatmap in (**B**) shows PERMANOVA R^2^ values for all separate variables that were added to the models as well as all interactions between variables with an R^2^ $\geq$ 0.05 and/or with *p* $\leq$ 0.05 (denoted with an asterisk). The columns show PERMANOVA tests for Progression (P/NP), Progression (grouped; NP and P grouped to <1, 1-2, 2-3, 3-4, 4-6 or 6+ years for time to progression) and Progressors only with the specific follow-up time. (**C**)-(**H**) prevalence and abundance for the top 30 most abundant species. (**C**) Phylogenetic tree showing the class of each species, and (**D**) a heatmap showing mean prevalence (blue scale). (**E**) and (**G**) show mean relative and CLR abundance, respectively, of species in progressor (P) or non-progressor (NP) samples. (F) and (H) are boxplots showing relative and CLR abundance, respectively, in all samples (right). Numbers below the relative abundance heatmap indicate the relative abundance accounted for in this plot. In the boxplots, each sample is shown as an individual point and boxes show the median, upper and lower quartiles while whiskers show the range of the data (1.5 times the interquartile range). |
| --- |

| 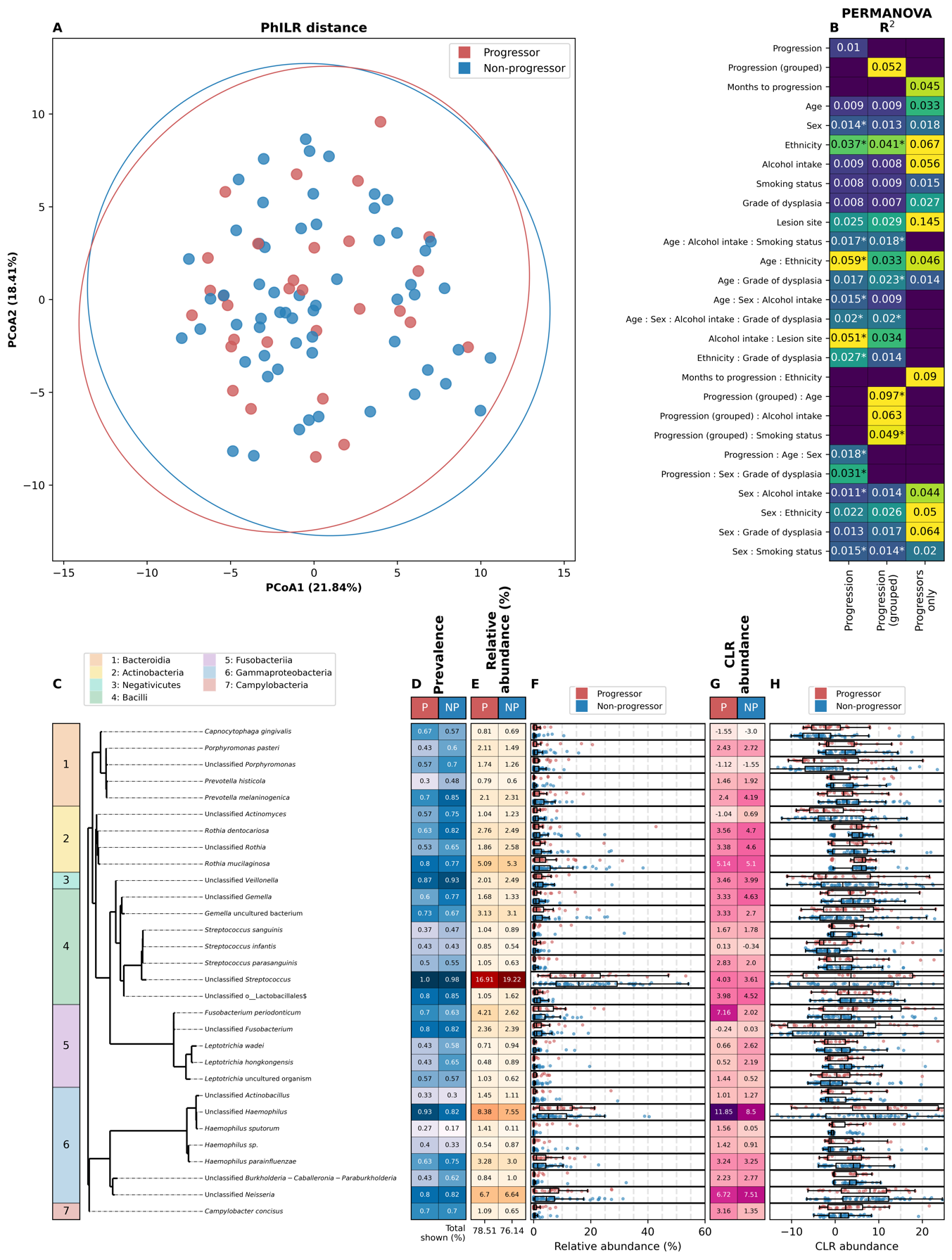  **Supp. Figure 2.** Sample diversity at the species level with taxonomic classifications from the SILVA database. (**A**) Principal Coordinates Analysis (PCoA) and (B) PERMANOVA tests using Phylogenetic Isometric Log Ratio (PhILR) distance. Ellipses show the confidence interval (3 standard deviations) for each group and the values shown on each axis label indicate the proportion of sample variation accounted for by that axis. The heatmap in (**B**) shows PERMANOVA R^2^ values for all separate variables that were added to the models as well as all interactions between variables with an R^2^ $\geq$ 0.05 and/or with *p* $\leq$ 0.05 (denoted with an asterisk). The columns show PERMANOVA tests for Progression (P/NP), Progression (grouped; NP and P grouped to <1, 1-2, 2-3, 3-4, 4-6 or 6+ years for time to progression) and Progressors only with the specific follow-up time. (**C**)-(**H**) prevalence and abundance for the top 30 most abundant species. (**C**) Phylogenetic tree showing the class of each species, and (**D**) a heatmap showing mean prevalence (blue scale). (**E**) and (**G**) show mean relative and CLR abundance, respectively, of species in progressor (P) or non-progressor (NP) samples. (F) and (H) are boxplots showing relative and CLR abundance, respectively, in all samples (right). Numbers below the relative abundance heatmap indicate the relative abundance accounted for in this plot. In the boxplots, each sample is shown as an individual point and boxes show the median, upper and lower quartiles while whiskers show the range of the data (1.5 times the interquartile range). |
| --- |

| 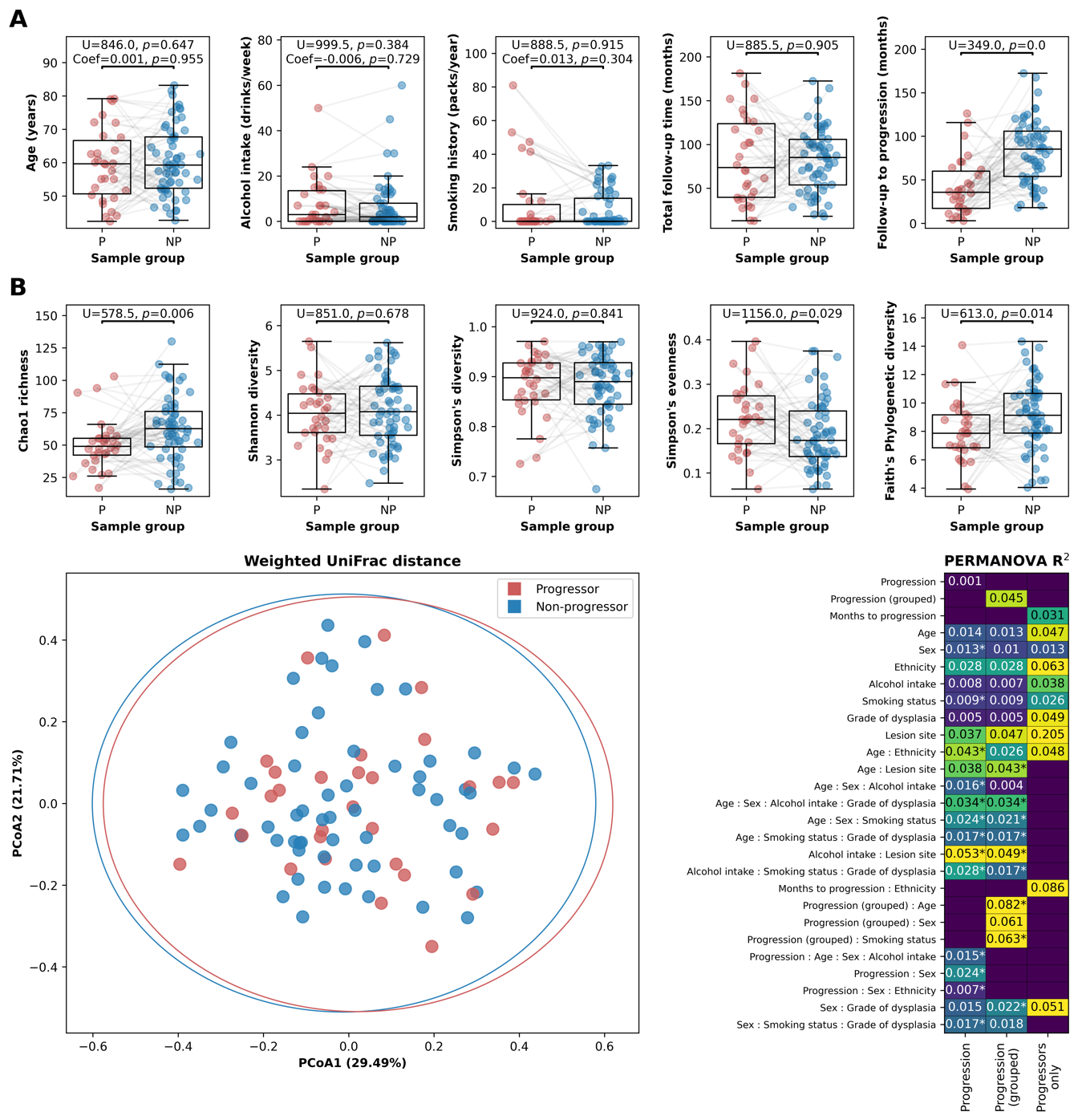  **Supp. Figure 3. S**ample diversity at the species level. Principal Coordinates Analysis (PCoA) and PERMANOVA tests using Weighted UniFrac distance (with rarefied count tables; **Supp. Table 1C**). Ellipses show the confidence interval (3 standard deviations) for each group and the values shown on each axis label indicate the proportion of sample variation accounted for by that axis. The heatmap shows PERMANOVA R^2^ values for all separate variables that were added to the models as well as all interactions between variables with an R^2^ $\geq$ 0.05 and/or with *p* $\leq$ 0.05 (denoted with an asterisk). All PERMANOVA R^2^ and *p* values are shown in **Supp. Table 1C**. The columns show PERMANOVA tests for Progression (P/NP), Progression (grouped; NP and P grouped to <1, 1-2, 2-3, 3-4, 4-6 or 6+ years for time to progression) and Progressors only with the specific follow-up time. |
| --- |

| 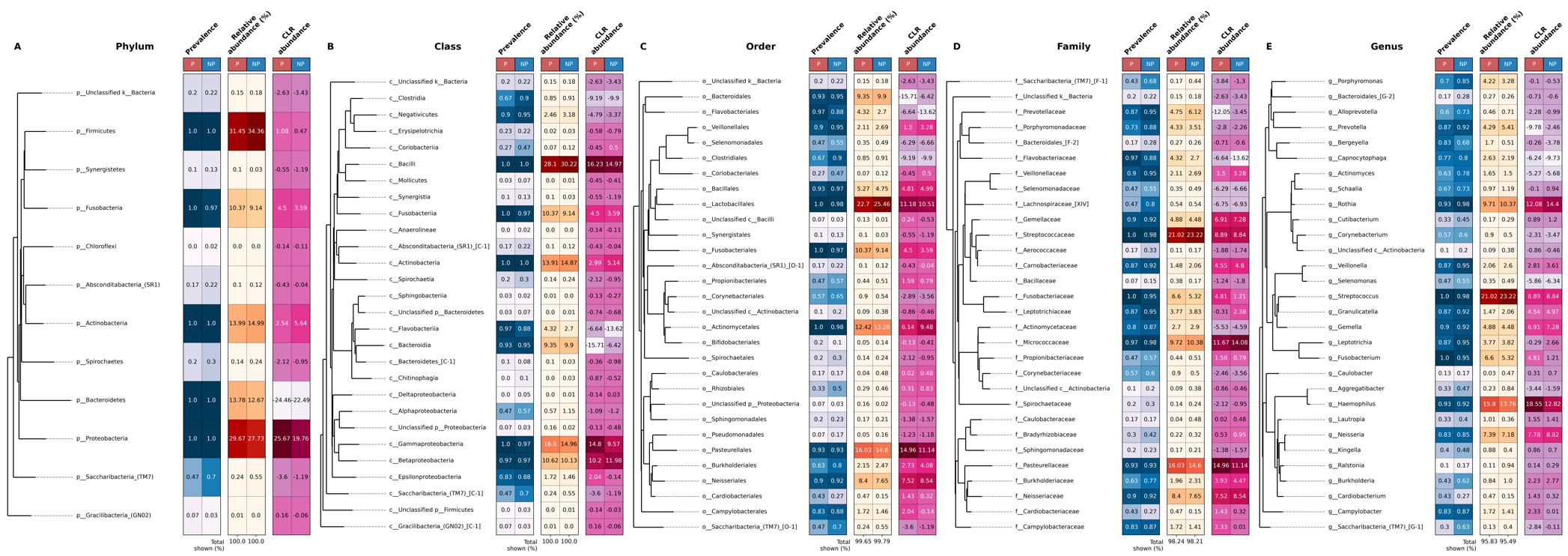  **Supp. Figure 4.** Mean prevalence and abundance of taxa at the Phylum (**A**), Class (**B**), Order (**C**), Family (**D**), or Genus (**E**) levels. Heatmaps show mean prevalence (blue scale; left), relative abundance (red scale; centre) and CLR abundance (purple scale; right) of taxa in progressor (P) and non-progressor (NP) samples while phylogenetic trees are shown to the left of each set of heatmaps. Numbers below the relative abundance heatmap indicate the relative abundance accounted for in each plot. Only the top 30 most abundant taxa are shown for each phylogenetic level. |
| --- |

| \| ~~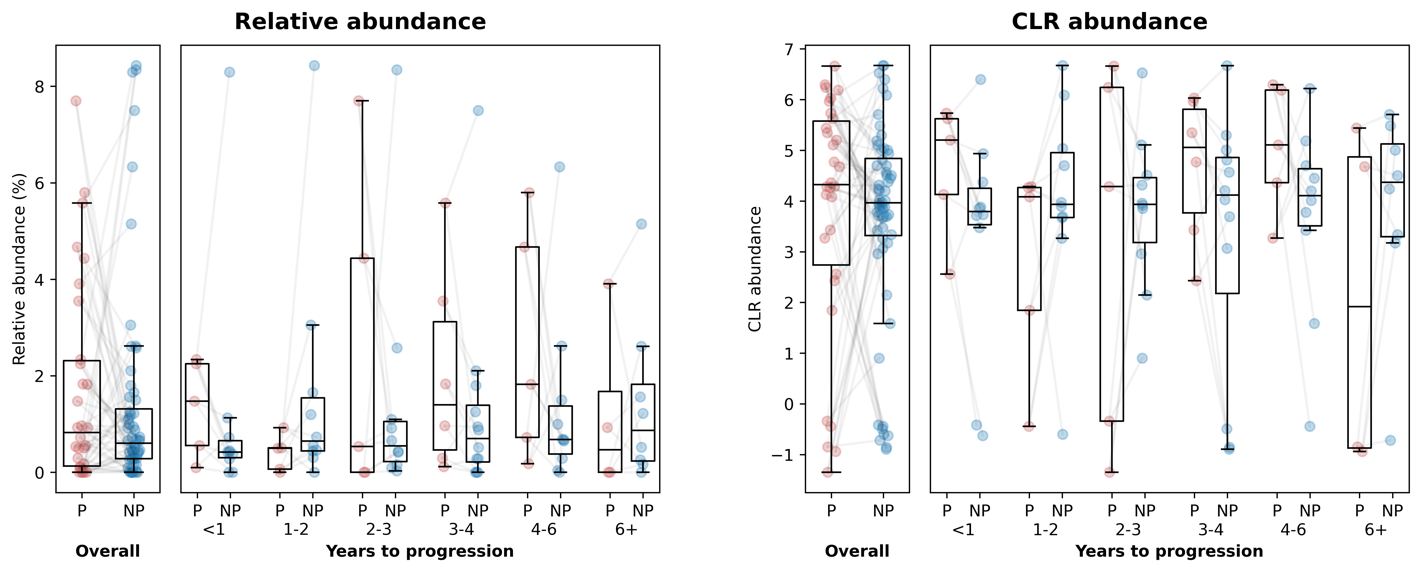~~ **Supplementary Figure 5.** Abundance of *Campylobacter* in Ps and NPs. Relative (left two plots) and CLR (right two plots) abundance of the *Campylobacter* genus (found to be significantly differentially abundant between Ps, grouped to time to progression, and NPs by MaAsLin2 tests with CLR abundance and with or without the other clinical variables) with samples grouped Ps and NPs overall or by the number of years to progression. In the boxplots, each sample is shown as an individual point and boxes show the median, upper and lower quartiles while whiskers show the range of the data (1.5 times the interquartile range). \| \| --- \| |
| --- | --- |

| 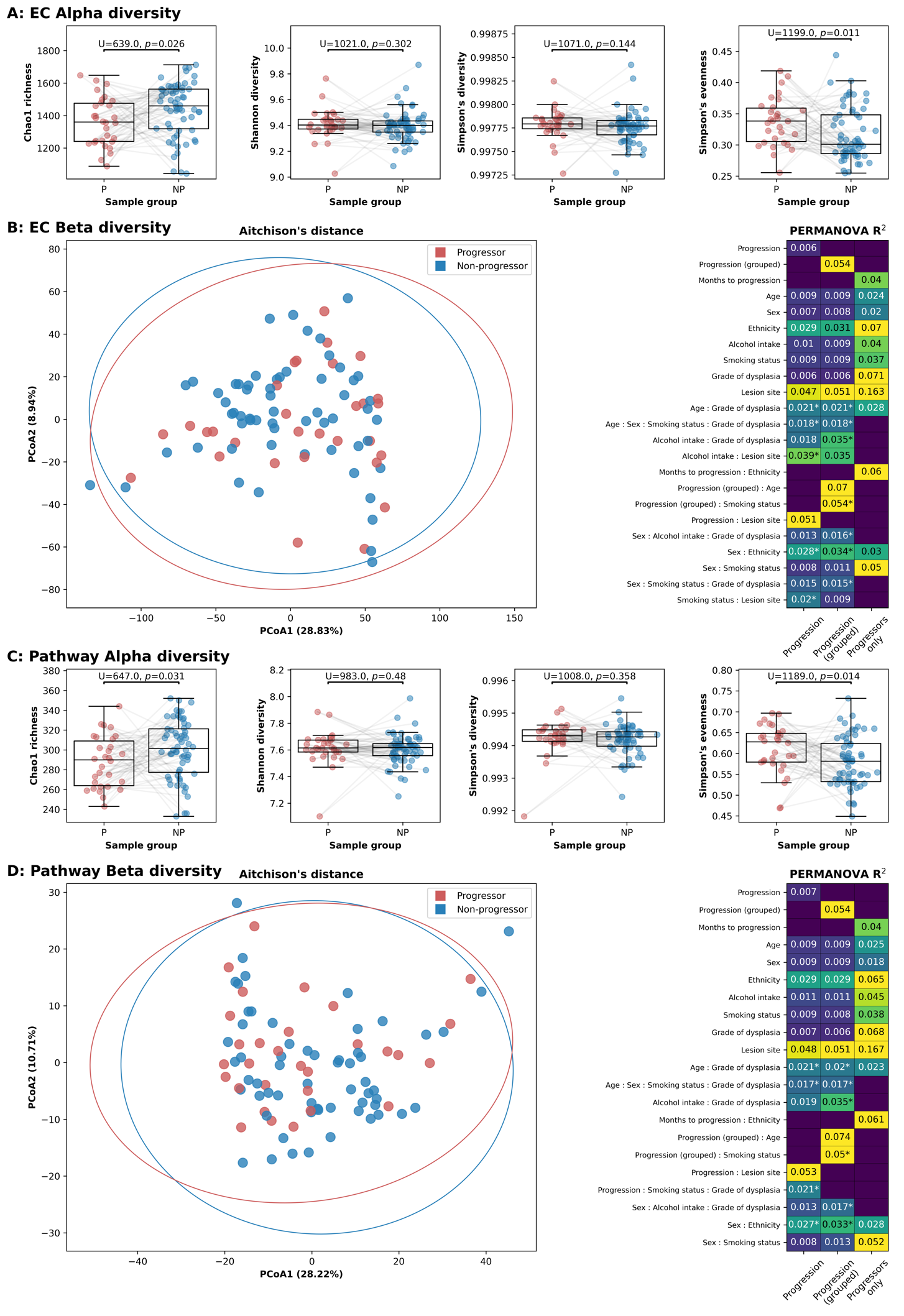  **Supplementary Figure 6.**  Diversity of sample PICRUSt2 profiles. (**A**) and (**B**) show data for functional profiles at the Enzyme Commission (EC) number level and (**C**) and (**D**) show functional profiles at the MetaCyc Metabolic pathway level. (**A**) and (**C**) Functional diversity within the microbial communities of 30 progressing (P) and 60 non-progressing (NP) oral epithelial dysplasia (OED) samples using four different alpha-diversity metrics: Chao1 richness, Shannon diversity, Simpson’s diversity, and Simpson’s evenness. U and *p*-values in (**A**) and (**C**) were determined by Mann-Whitney U tests and lines indicate matched Ps and NPs. Lines between points indicate matched Ps/NPs and boxes show the median, upper and lower quartiles while whiskers show the range of the data (1.5 times the interquartile range). (**B**) and (**D**) Principal Coordinates Analysis (PCoA) and PERMANOVA tests using Aitchison’s distance. Ellipses show the confidence interval (3 standard deviations) for each group and the values shown on each axis label indicate the proportion of sample variation accounted for by that axis. The heatmaps in (**B**) and (**D**) show PERMANOVA R^2^ values for all separate variables that were added to the models as well as all interactions between variables with an R^2^ $\geq$ 0.05 and/or with *p* $\leq$ 0.05 (denoted with an asterisk). The columns show PERMANOVA tests for Progression (P/NP), Progression (grouped; NP and P grouped to <1, 1-2, 2-3, 3-4, 4-6 or 6+ years for time to progression) and Progressors only with the specific follow-up time. |
| --- |

| 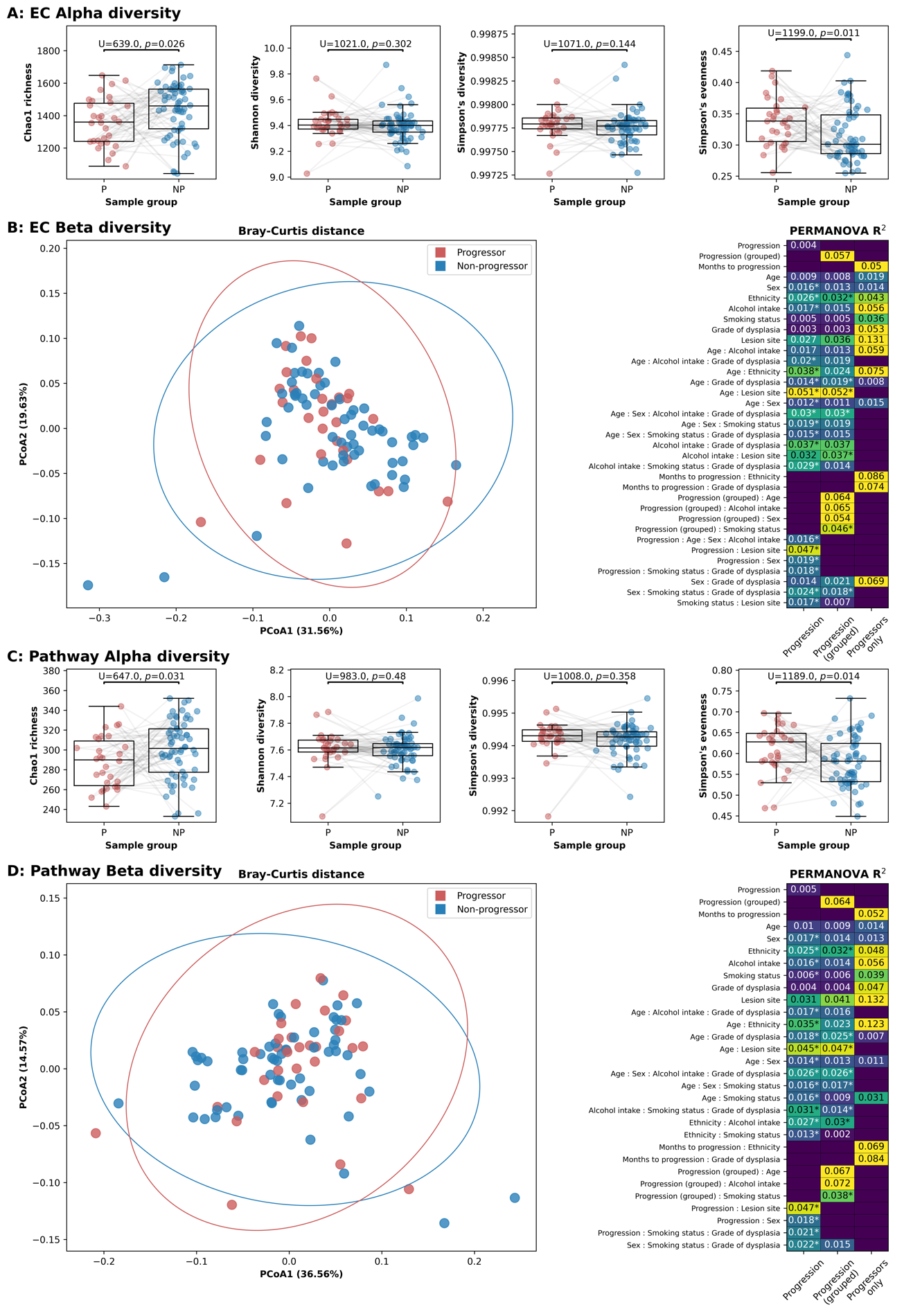  **Supplementary Figure 7.**  Diversity of sample PICRUSt2 profiles. (**A**) and (**B**) show data for functional profiles at the Enzyme Commission (EC) number level and (**C**) and (**D**) show functional profiles at the MetaCyc Metabolic pathway level. (**A**) and (**C**) Functional diversity within the microbial communities of 30 progressing (P) and 60 non-progressing (NP) oral epithelial dysplasia (OED) samples using four different alpha-diversity metrics: Chao1 richness, Shannon diversity, Simpson’s diversity, and Simpson’s evenness. U and *p*-values in (**A**) and (**C**) were determined by Mann-Whitney U tests and lines indicate matched Ps and NPs. Lines between points indicate matched Ps/NPs and boxes show the median, upper and lower quartiles while whiskers show the range of the data (1.5 times the interquartile range). (**B**) and (**D**) Principal Coordinates Analysis (PCoA) and PERMANOVA tests using Bray-Curtis distance. Ellipses show the confidence interval (3 standard deviations) for each group and the values shown on each axis label indicate the proportion of sample variation accounted for by that axis. The heatmaps in (**B**) and (**D**) show PERMANOVA R^2^ values for all separate variables that were added to the models as well as all interactions between variables with an R^2^ $\geq$ 0.05 and/or with *p* $\leq$ 0.05 (denoted with an asterisk). The columns show PERMANOVA tests for Progression (P/NP), Progression (grouped; NP and P grouped to <1, 1-2, 2-3, 3-4, 4-6 or 6+ years for time to progression) and Progressors only with the specific follow-up time. |
| --- |

| 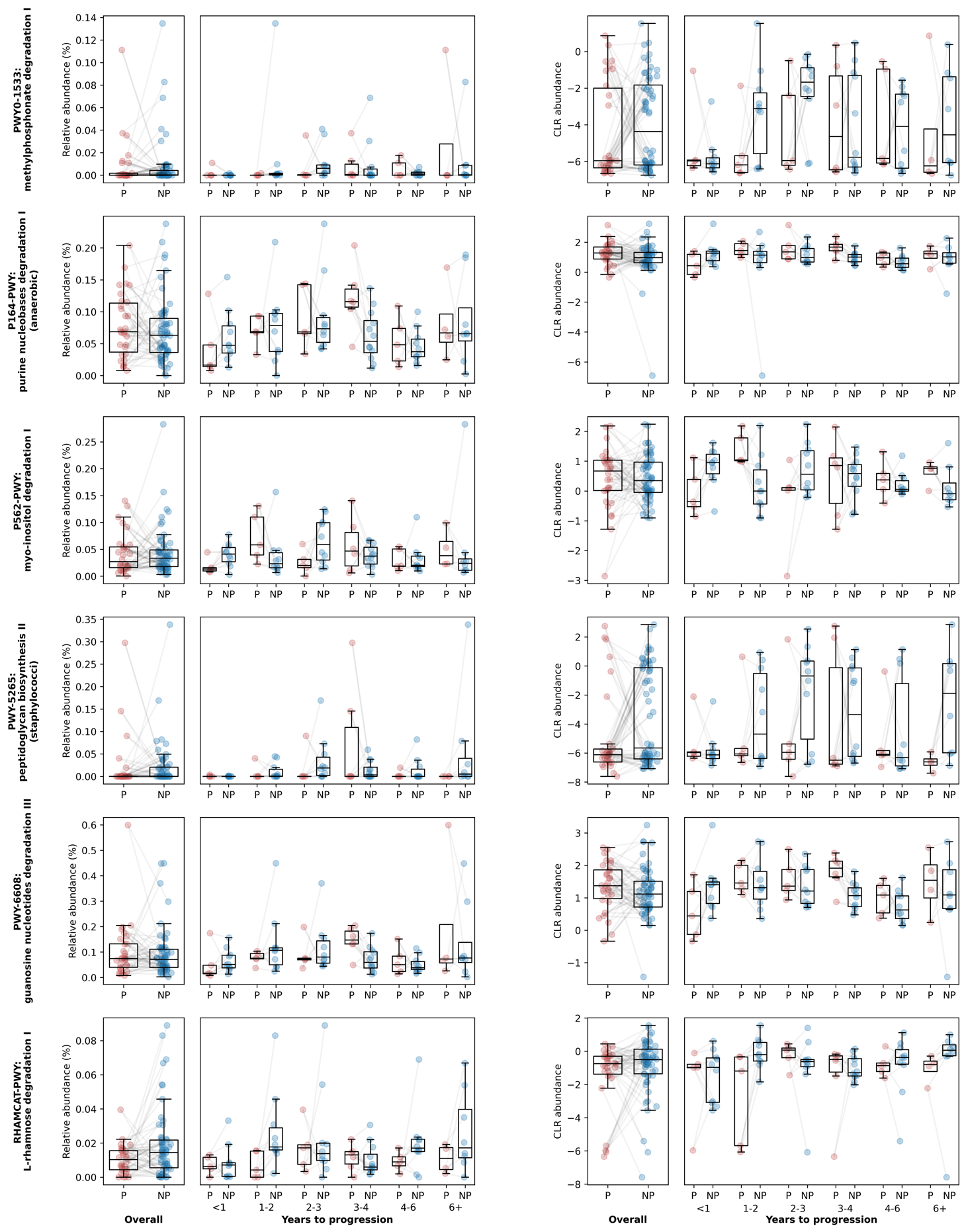  **Supplementary Figure 8.** Abundance of pathways found to be significantly differentially abundant by MaAsLin2 tests between Ps and NPs with CLR abundance and both with and without the inclusion of clinical variables. Each row shows a different pathway, with the left two plots showing relative abundance and the right two showing CLR abundance. In the boxplots, each sample is shown as an individual point and boxes show the median, upper and lower quartiles while whiskers show the range of the data (1.5 times the interquartile range). Grey lines show the matched Ps and NPs. The top pathway (PWY-01553) was found to be significantly differentially abundant between Ps and NPs (overall), while all other pathways were found to be significantly differentially abundant between Ps grouped to time to progression and NPs. |
| --- |

| 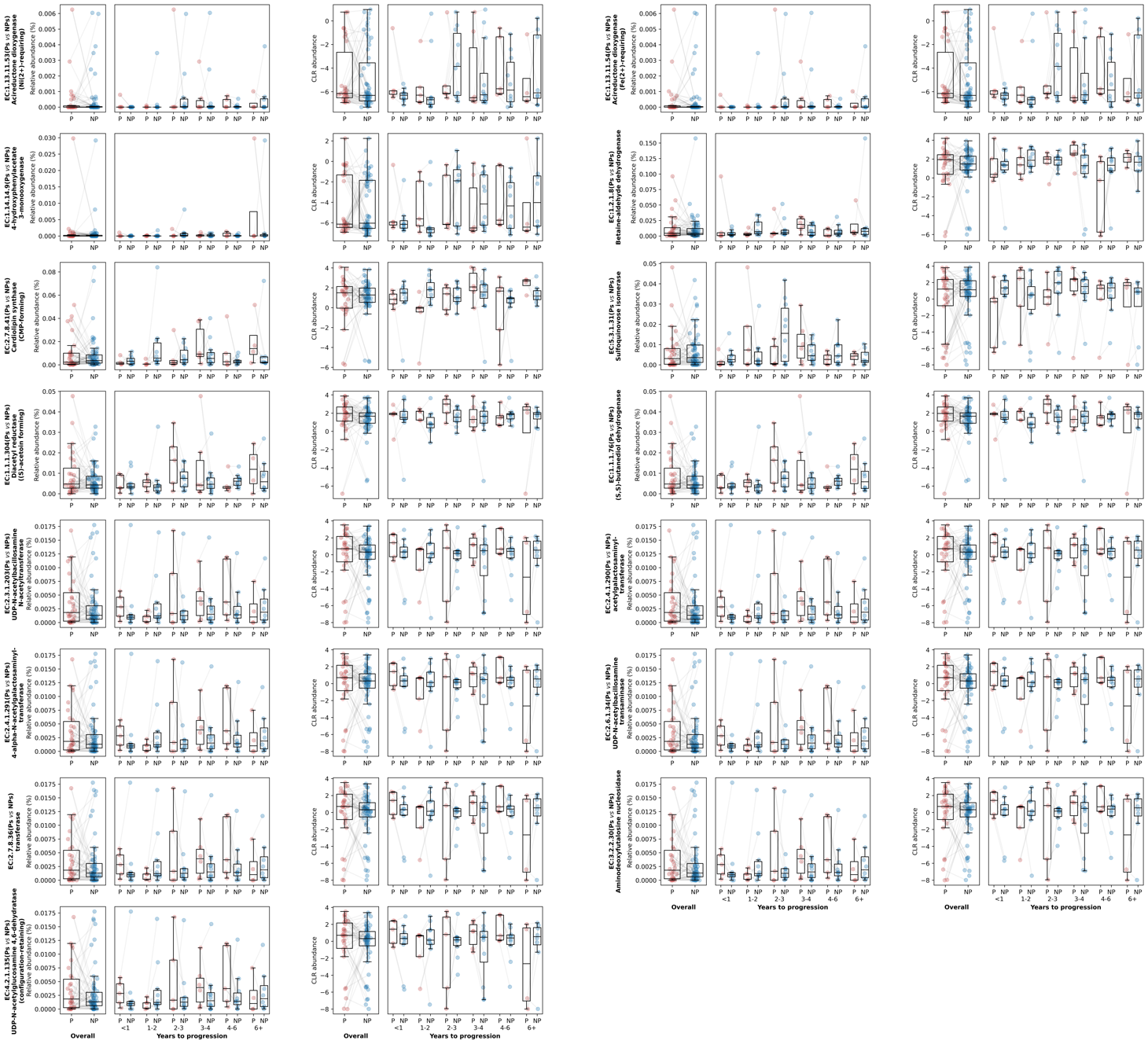  **Supplementary Figure 9.** Relative and CLR abundance for the 15 EC numbers (predicted by PICRUSt2) that were identified as differentially abundant with progression status by MaAsLin2. For each enzyme (EC number), the columns show relative (first two columns) or CLR abundance (second two columns) Ps (red) and NPs (blue) overall (left) or for Ps grouped to time to progression and NPs (right). Each point represents a single sample, lines between points indicate matched Ps/NPs and boxes show the median, upper and lower quartiles while whiskers show the range of the data (1.5 times the interquartile range). |
| --- |

| **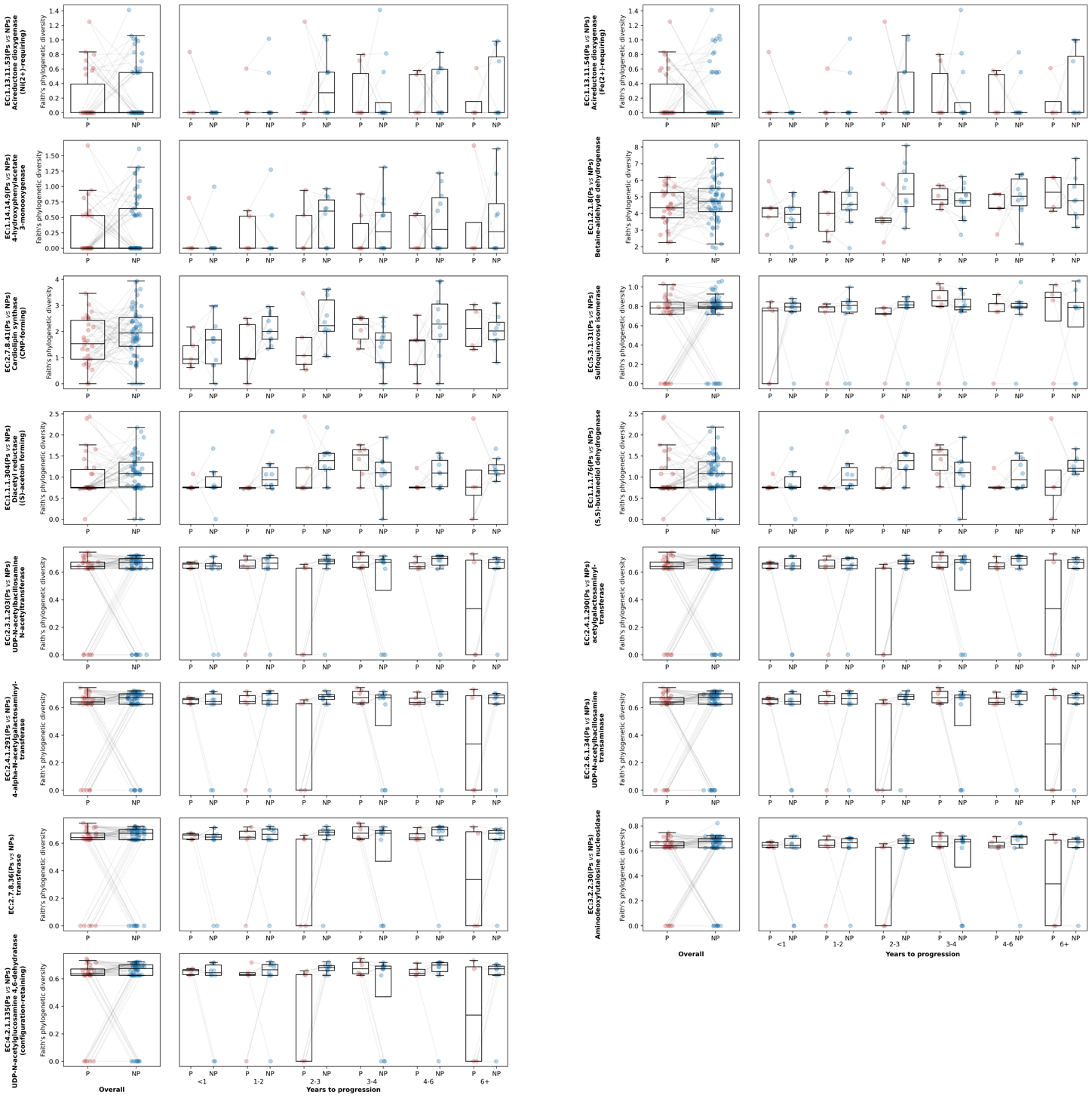**  **Supplementary Figure 10.** Contributional diversity for the 15 EC numbers (predicted by PICRUSt2) that were identified as differentially abundant with progression status by MaAsLin2. Contributional diversity refers to the diversity of the ASVs that possess that enzyme (EC number). For each enzyme (EC number), the columns show Faith’s phylogenetic diversity of the ASVs that possess that enzyme for Ps (red) and NPs (blue) overall (left) or for Ps grouped to time to progression and NPs (right). Each point represents a single sample, lines between points indicate matched Ps/NPs and boxes show the median, upper and lower quartiles while whiskers show the range of the data (1.5 times the interquartile range). |
| --- |

**References**

1. Quast C, Pruesse E, Yilmaz P, Gerken J, Schweer T, Yarza P, et al. The SILVA ribosomal RNA gene database project: improved data processing and web-based tools. Nucleic Acids Res. 2013 Jan;41(Database issue):D590-596.

2. Chen T, Yu WH, Izard J, Baranova OV, Lakshmanan A, Dewhirst FE. The Human Oral Microbiome Database: a web accessible resource for investigating oral microbe taxonomic and genomic information. Database. 2010 Jul 30;2010(0):baq013–baq013.

3. Bokulich NA, Kaehler BD, Rideout JR, Dillon M, Bolyen E, Knight R, et al. Optimizing taxonomic classification of marker-gene amplicon sequences with QIIME 2’s q2-feature-classifier plugin. Microbiome. 2018 Dec;6(1):90.
